# ATF6 Promotes Colorectal Cancer Growth and Stemness by Regulating the Wnt Pathway

**DOI:** 10.1158/2767-9764.CRC-24-0268

**Published:** 2024-10-21

**Authors:** Jeffrey J. Rodvold, Matthew Grimmer, Karen Ruiz, Scot A. Marsters, Ioanna Oikonomidi, Eileen Tan-Aristy, Victoria C. Pham, Tamal Sarkar, Jonathan M. Harnoss, Whitney Shatz-Binder, Zora D. Modrusan, Thomas D. Wu, Jennie R. Lill, Elisia Villemure, Joachim Rudolph, Felipe de Sousa e Melo, Avi Ashkenazi

**Affiliations:** 1 Department of Research Oncology, Genentech, Inc., South San Francisco, California.; 2 Department of Computational Science, Genentech, Inc., South San Francisco, California.; 3 Department of Discovery Oncology, Genentech, Inc., South San Francisco, California.; 4 Department of Microchemistry, Proteomics, and Lipidomics, Genentech, Inc., South San Francisco, California.; 5 Department of General, Visceral, Thoracic, and Transplantation Surgery, University Hospital Giessen, Giessen, Germany.; 6 Department of Pharmaceutical Development, Genentech, Inc., South San Francisco, California.; 7 Department of Discovery Chemistry, Genentech, Inc., South San Francisco, California.

## Abstract

The unfolded protein response maintains endoplasmic reticulum homeostasis by sensing protein-folding stress and orchestrating cellular adaptation via the endoplasmic reticulum transmembrane proteins IRE1, PERK, and ATF6. Malignant cells can co-opt IRE1 and PERK to sustain growth; however, the importance of ATF6 in cancer remains poorly deciphered. We observed elevated ATF6 transcriptional activity in several cancers, including colorectal carcinoma. Genetic silencing or small-molecule inhibition of ATF6 blocked cell-cycle progression and reduced viability of several human colorectal cancer cell lines *in vitro*, and disrupted tumor progression *in vivo*. Unexpectedly, ATF6 interference disabled Wnt and Myc signaling and reduced stemness. ATF6 inhibition attenuated growth of organoids derived from malignant but not normal human intestinal tissue, decreasing Wnt pathway activity and driving cellular differentiation. Wnt-surrogate agonism in a Wnt ligand-dependent colorectal cancer organoid restored pathway activity and rescued growth under ATF6 blockade. Our findings uncover ATF6 as an unexpected facilitator of oncogenic Wnt signaling in colorectal cancer.

**Significance:**

ATF6 intervention reduces colorectal cancer cell and organoid viability by interrupting dysregulated Wnt signaling, identifying a novel facilitator and potential therapeutic target in colorectal cancer.

## Introduction

Colorectal cancer is among the most globally prevalent and deadly forms of malignancy (1) and is now the leading cause of cancer-related mortality in individuals under 50 years old (2). Despite refined screening techniques (3), late-stage colorectal cancer continues to have poor outcomes. Successful singular or combinatorial treatments have been developed to intervene against several colorectal cancer driver mutations, including in *BRAF*, *KRAS*, and *EGFR* (4). In addition, although mutations in the Wnt pathway are much more prevalent, occurring in nearly 95% of colorectal cancer cases, strategies to intervene against oncogenic Wnt signaling have yet to yield a meaningful clinical breakthrough (5–7).

Dysregulated Wnt signaling confers a number of tumorigenic cellular properties (8), including increased MYC expression and activity (9), uncontrolled cell-cycle progression (10), and sustained stemlike cellular function (11). In the context of cancer, stemlike features permit a small number of seeding cells to establish and sustain both primary and metastatic lesions. Wnt pathway target genes such as *LGR5* (12) and *CD44* (13) efficiently mediate stemness. The ablation of LGR5^+^ in intestinal cells limits tumor growth and metastasis; however, these events can resume through positive selection of cells that have regained LGR5 expression (14). Stemlike cell populations are considered especially resistant to traditional forms of cancer therapy (15). Given the central role of Wnt in colorectal cancer and the challenges to date in attaining effective Wnt intervention, an alternative strategy toward discovery of new treatments is to identify orthogonal vulnerabilities that disable the Wnt pathway.

One intrinsic cell state that reportedly opposes stem signaling is endoplasmic reticulum (ER) stress (16–18). The ER is a key cellular organelle that mediates the folding of most transmembrane and secreted proteins. Imbalances in ER homeostasis, often caused by excessive protein biosynthetic burden or disruptions in protein folding, lead to ER stress. To mitigate ER stress, eukaryotic cells leverage the unfolded protein response (UPR), which is coordinated by three stress-responsive ER transmembrane proteins: IRE1, PERK, and ATF6. Cancer cells can co-opt specific aspects of the UPR to accommodate the unsatiable demands of oncogene-driven growth. For example, BRAF^V600E^ activates IRE1 and ATF6 (19), whereas oncogenic Myc drives IRE1 transcription (20) to support malignant proliferation. Chromosomal instability also engages UPR activity (21) as does infection by viruses (22, 23), including recognized oncogenic strains such as hepatitis C (24). Apart from cell-intrinsic drivers, the tumor microenvironment harbors many cell-extrinsic insults that cause ER stress, including hypoxia (25), reactive oxygen species (26), and nutrient scarcity (27). Whereas numerous reports ascribe tumor-promoting roles to IRE1 (28, 29) and PERK (30, 31), the potential role of ATF6 in supporting malignancy is much less established (32).

Unlike its sister UPR branches, ATF6 does not act as an ER-resident enzyme but rather operates as a latent transcription factor, similar to sterol regulatory element-binding proteins (33). There are two human ATF6 paralogs—ATF6α (herein ATF6), and ATF6β—both of which are ubiquitously expressed in tissues. Upon ER stress, the ER lumenal domain of oligomerized ATF6 becomes dissociated, unmasking localization sequences that facilitate ATF6 trafficking to the Golgi compartment through Coat Protein Complex II (COPII) vesicles. Two Golgi-resident proteases then process ATF6 to produce a 50 kDa cytoplasmic fragment—nuclear ATF6 (nATF6; ref. 34), which relocates to the nucleus to act as a transcription factor. In contrast to ATF6α, a clear transcriptional role for ATF6β has yet to be established (35). nATF6 activates an incompletely defined set of genes that includes chaperones and protein disulfide isomerases, believed to help restore proteostasis (35). In this study, we show that ATF6 promotes colorectal cancer through an unexpected yet critical role in facilitating oncogenic Wnt signaling.

## Materials and Methods

### Methods for ER13 signature scores

The selected ER13 genes were first described in Shoulders and colleagues (36). Specifically, the ectopic expression of nATF6 bound to a destabilized variant of DHFR increased the expression of 13 genes in transduced HEK293T-REx cells. Of this gene cohort, only five genes were also induced by ectopic expression of spliced XBP1 (XBP1s). Induced genes that required the simultaneous ectopic expression of nATF6 and XBP1s were excluded from ER13, as the cooperativity of the cofactors implied necessary activation of *both* ATF6 and IRE1 branches. Of the considered 13 genes, 2 were also present in our previous ER16 analysis (*HERPUD1* and *PDIA6*; ref. 29). The ER13 gene set, as well as the ER16 for comparison, are included in Supplementary Table S1.

For pan-cancer analysis, FASTQ RNA sequencing (RNA-seq) files were obtained from The Cancer Genome Atlas (TCGA) and processed through a standard pipeline using the HTSeqGenie package (37) in BioConductor (38), as follows. First, reads with low nucleotide qualities (70% of bases with quality <23) or matches to ribosomal RNA and adapter sequences were removed. The remaining reads were aligned to the human reference genome (NCBI build 38) using GSnap (39) version 2023-10-10-v2 with parameters “-M 2 -n 10 -B 2 -i 1 -N 1 -w 200000 -E 1 –pairmax-rna = 200,000 –clip-overlap”, which allowed a maximum of two mismatches per 75-bp sequence. Transcript annotation was based on Ensembl release 77 (RRID: SCR_002344). Reads per kilobase per million mapped reads (RPKM) values were obtained by multiplying the alignment counts in each library by the size factor computed from the DESeq2 package (RRID: SCR_000154) for the genes *HSPA5* (Ensembl Gene ID 3309), *HSP90B1* (7184), *CALR* (811), *PDIA4* (9601), *ERO1A*/*ERO1L* (30001), *HERPUD1* (9709), *OS9* (10956), *SEL1L* (6400), *DNAJB11*/*ERDJ3* (51726), *PDIA3*/*ERP57* (2923), *PDIA6* (10130), *UGGT1* (56886), and *VCP* (7415). For each gene, a *Z*-score was computed by subtracting its mean and dividing by its SD over all samples in the dataset. A signature score for ER13 was computed by taking the mean of these *Z*-scores over all 13 genes in the signature. Sample types were obtained from the barcode for each sample using the definitions at http://gdc.cancer.gov/resources-tcga-users/tcga-code-tables/sample-type-codes to assign samples as being cancer (primary tumor or metastatic) or normal. Statistical comparisons between cancer and normal samples, when available for a given organ, were made using the *t* test function in R to compute a Student two-sided *t*-test statistic.

Annotation of colon cancer samples in TCGA was obtained from the Genomic Data Commons data portal at https://portal.cdc.cancer.gov/projects/TCGA-COAD and by downloading the clinical information. Samples were joined using TCGA sample barcodes and the case_submitter_id field in the clinical annotation. Samples were grouped using the primary_diagnosis field in the clinical annotation. Statistical comparisons between cancer and normal samples were made using the *t* test function in R to compute a Student two-sided *t*-test statistic.

### ATF6 and ER13 signature expression analysis in single-cell RNA-seq data

Publicly available single-cell RNA-seq (scRNA-seq) data from human colorectal tumors and adjacent normal tissues were analyzed for the expression of ATF6 and the ER13 gene signature in different cell subtypes (40). The nomenclature of the clusters was used as described in the original article. Briefly, the CLOUPE file was loaded for visualization and analysis using the loupe browser v7.0 (https://www.10xgenomics.com/support/software/loupe-browser/latest). Transformation of raw counts of gene expression to log scale allowed for further expression analysis. For ATF6 expression analysis, the log_2_ expression value was assessed, whereas for the ER13 signature, the log_2_ average expression levels was taken. The microsatellite (MS) status was obtained from the original article, and ER13 expression was compared between cells from normal samples and tumor samples based on the MS stability status.

### Cell culture and treatments

All cell lines were obtained or generated from an internal repository maintained at Genentech, which were authenticated by short tandem repeat profiles. Cells were tested to ensure they were *Mycoplasma*-free within 3 months of use. All lines were maintained at low passage numbers and were not kept after 15 passages from thawing. Cell lines were cultured in RPMI 1640 media supplemented with 10% (v/v) FBS (Sigma), 2 mmol/L glutaMAX (Gibco), 100 U/mL penicillin (Gibco), and 100 μg/mL streptomycin (Gibco) in a 5% CO_2_ incubator at 37°C.

For organoid culture, PDM-264 (HCM-CSHL-0382-C19), PDM-5 (HCM-CSHL-0061-C18), PDM-6 (HCM-CSHL-0062-C18), PDM-96 (HCM-CSHL-0143-C20), PDM-185 (HCM-CSHL-0238-C18), and PDM-272 (HCM-CSHL-0459-C17) were initially obtained from the ATCC and maintained in Organoid Media Formulation #1 (ATCC). Specifically, the formulation using advanced DMEM:F12 is supplemented with 10 mmol/L 4-(2-hydroxyethyl)-1-piperazine ethanesulfonic acid (HEPES), 2 mmol/L L-glutamine, 1× B27 supplement (Thermo Fisher Scientific), 100 ng/mL Noggin, 50 ng/mL EGF, 10 nmol/L gastrin (Tocris), 10 μmol/L SB202190 (Selleckchem), 500 nmol/L A83-01 (Tocris), 10 mmol/L nicotinamide (Sigma), and 1.25 mmol/L N-acetyl cysteine. This formulation does not contain supplemented Wnt ligands. Col009 and DNW15 were derived from healthy intestinal tissue and maintained as previously described (41) using media supplemented with WNT3a. All organoids were cultured using Matrigel Growth Factor Reduced Basement Membrane Matrix and Phenol Red-free (Corning) plugs mixed evenly with 50:50 with organoid media. Organoids were dissociated using Accutase (StemCell) and physical dissociation through pipetting.

Treatments included thapsigargin (Tg; 100 nmol/L, Sigma,), doxycycline (Dox; 0.5 μg/mL, Takara) pan-caspase inhibitor z-VAD-FMK (zVAD; 20 μmol/L, Selleckchem), and Wnt surrogate-FC fusion recombinant protein (Thermo Fisher Scientific) at specified concentration and time. Ceapin-A7 and inactive Ceapin-A7 were synthesized in-house.

### shRNA knockdown of ATF6, IRE1, and β-catenin

Parental Colo201, CCK81, Colo205, LS411N, HT55, SW1463 (RRID: CVCL_1718), SW48 (RRID: CVCL_1724), SW480 (RRID: CVCL_0546), Colo320DM, LS186, HCT116 (RRID: CVCL_0291), SNUC1, SW403 (RRID: CVCL_0545), and RKO (RRID:CVCL_0504) cell lines were transfected with a Dox-inducible plasmid to simultaneously express three different *ATF6* short hairpin RNAs (shRNA) or one shRNA for nontargeting control (NTC; see sequences below). ATF6 shRNAs were blasted to confirm that no predicted gene except *ATF6* was significantly targeted (Supplementary Table S2). Each shRNA was cloned in the piggyBac Dox-inducible ODE vector containing a puromycin resistance marker. TransIT-X2 Dynamic Delivery System (Mirus) was used for transfection following the manufacturer’s instructions. Positively transfected cells were selected with puromycin (1–5 μg/mL). Surviving cells were cultured from single-cell clones, as specified using “0.1” nomenclature (e.g., “shATF6.1”) or as a pooled culture (“shATF6”) according to the knockdown efficiency, which was assessed by immunoblotting and/or qPCR. Similar approaches were used to establish shNTC, sh*ERN1* (IRE1α), and sh*CTNNB1* (β-catenin) in specified cell lines:

shNTC: 5′-TAG​ATA​AGC​ATT​ATA​ATT​CCT-3′

shATF6-2: 5′-TAG​AAG​ACA​AAT​CCA​ACT​CCT-3′

shATF6-4: 5′-TTT​GAG​TCT​TGG​GTG​CTG​CTG-3′

shATF6-6: 5′-TAC​GTT​GCT​GTC​TCC​TTA​GCA-3′

shIRE1-7: 5′-AGA​ACA​AGC​TCA​ACT​ACT​T-3′

shIRE1-8: 5′-GCA​CGT​GAA​TTG​ATA​GAG​A-3′

shIRE1-9: 5′-GAG​AAG​ATG​ATT​GCG​ATG​G-3′

shCTNNB1-1: 5′-TAG​CGT​GTC​TGG​AAG​CTT​CCT -3′

shCTNNB1-2: 5′- TTC​GGT​TGT​GAA​CAT​CCC​GAG-3′

shCTNNB1-3: 5′- TTCGGTTGTGAACARCCCG -3′

Blast analysis for ATF6 shRNA guides indicated no potential off targets having an e-score of <1 for shATF6-2 and shATF6-6. For shATF6-4, *ATF6* had an e-score of 0.008, whereas *MAP2K3* had an e-score of 0.13 (Supplementary Table S2). RNA-seq analysis confirmed silencing of *ATF6* but not *MAP2K3* in cells expressing all three ATF6 shRNAs (Supplementary Fig. S3I).

### CRISPR/Cas9 knockout of ATF6

Colo201 ATF6 knockout (KO) cells were generated from parental Colo201 using CRISPR technology by cotransfecting a Cas9-containing plasmid, pRK-TK-Neo-Cas9, with a ATF6 (*ATF6*) targeting guide RNA (5′-G TTG​CCA​ATG​GCA​TAA​GCG​T-3′) cloned into a pLKO vector (RRID: Addgene_139470). Colo201 cells were transfected using TransIT-X2 Dynamic Delivery System (Mirus) according to the manufacturer’s protocol, and FACS sorted the cells into single-cell clones based off of fluorescent positivity for positive expression. Single-cell clones with the most complete depletion of ATF6 were selected by immunoblotting.

### Antisense oligonucleotides

Two antisense oligonucleotides (ASO) against ATF6 were synthesized (IDT) and validated for ATF6 depletion. ATF6 and control ASOs were packaged using lipid nanoparticles (LNP; MC3/cholestrol/DPPC/DMG-PEG2K).

ATF6 ASO #1: ASO-1582: 5′-+T+T+TGA​GCC​CTG​TT+C+C+A-3′

ATF6 ASO #2: ASO-6986: 5′-+G+A+TAT​GCC​AAG​CC+T+C+C-3′

SNCA ASO: 5′-+A+T+TCCTTTACAC+C+A-3′

ATF6 and control ASOs were encapsulated in LNPs using the following formulation: 50% D-Lin-MC3-DMA, 38% cholesterol, 10% DPPC, and 2% DMG-PEG2K, with an N/P ratio of 2:1. All lipids were procured from Avanti Polar Lipids, Inc., except for D-Lin-MC3-DMA (Thermo Fisher Scientific). To characterize the final LNP formulations, dynamic light scattering (Wyatt Technology) was used to determine diameter and polydispersity, and Quant-iT OliGreen ssDNA Assay Kit (Thermo Fisher Scientific) was used to determine ASO concentration and encapsulation efficiency. Colo201 and HT29 cells were treated and analyzed as specified in text with LNPs.

### Cell confluency, viability, and caspase activity assays

Cells were seeded at 3 × 10^3^ to 10 × 10^3^ cells/well in clear, flat bottom 96-well plates (3595, Corning) for most viability studies. For ultra-low attachment (ULA) studies, cells were seeded at 3 × 10^3^ to 10 × 10^3^ cells/well in ULA 96-well plates (7007, Corning) and centrifuged at 1,000 *g* for 5 minutes to facilitate spheroid formation. Chemical treatments occurred after cell adherence, where appropriate, using complete RPMI media to a final working volume of 100 μL/well. Culture confluency was tracked using a live cell imaging system (Incucyte Zoom, Essen Bioscience), which calculates culture confluency as a percent (%) from the cell occupancy of the total well area from a generated image. These images were taken at a frequency of 3- or 4-hour intervals using a 4× or 10× objective. Confluency percent was then plotted as a function of time. Organoid confluence was similarly calculated using specific organoid analysis software (Incucyte Zoom, Essen Bioscience). Tissue culture (TC) cell viability was quantified from the culture’s ATP consumption using CellTiter-Glo Assay (Promega). In the case of ULA, Matrigel, and all organoid studies, CellTiter-Glo 3D Cell Viability Assay (Promega) was used. In both instances, analyses followed the manufacturer’s instructions. Luminescence was measured on an Envision system (PerkinElmer). To determine cellular viability as a percent (%), averaged raw luminescence units were normalized to the negative/untreated control condition.

### Subcutaneous xenograft growth and efficacy studies

All animals were maintained in accordance with the Guide for the Care and Use of Laboratory Animals (National Research Council, 2011). All animal studies were conducted under protocols approved by the Genentech Institutional Animal Care and Use Committee. Genentech is an AAALAC-accredited facility. Mice were housed in individually ventilated cages and maintained on a standard 14:10-hour light:dark cycle in temperature- (68°F–79°F) and humidity-controlled (30%–70%) rooms.

For Colo201 studies, including cell lines Colo201 shNTC, shATF6.1, shATF6.2, CRISPR ATF6 KO Cl 4.5, CRISPR ATF6 KO Cl 4.8, or parental Colo201 cell line, 1 × 10^6^ cells were suspended in 1:1 Hank’s Balanced Salt Solution and Matrigel (Corning) to a final volume of 100 μL and injected subcutaneously in the right flank of 6 to 8 weeks old female C.B-17 SCID mice. For CCK81 studies, including the shATF6 line, 5 × 10^6^ cells were inoculated in 6 to 8 weeks old female C.B-17 SCID mice. For inducible knockdown studies, when inoculated tumors reached ∼100 mm^3^, mice were randomized into one of two treatment groups: vehicle (5% sucrose drinking water, changed once per week) or Dox (0.5 mg/mL Dox in 5% sucrose drinking water, resupplemented 3 times per week). For tumor efficacy studies, at least 10 animals per group were treated, and tumor growth was monitored until the last vehicle control animal reached humane endpoint (tumor volume reached or exceeded 2,000 mm^3^). Tumor volume was calculated using the standard ellipsoid formula: volume = 0.523 × length × width × width. Mice were humanly euthanized according to signs of clinical pain or distress, significant body weight loss (>20%), or tumors exceeding a volume of 2,000 mm^3^ or presenting with ulceration.

### Immunoblot analysis

To prepare TC cells and organoids for immunoblotting, samples were first washed once with ice-cold PBS and pelleted. To prepare tumor specimens, tissues samples were first surgically isolated, placed on dry ice, and then mechanically disrupted using Bead Ruptor Elite (Omni). Samples were lysed with RIPA lysis buffer (20–188, Millipore) containing 2× Halt Protease and Phosphatase Inhibitor Cocktail (Thermo Fisher Scientific) and kept on ice for 15 to 30 minutes. Lysates were cleared by centrifugation at 14,000 *g* for 15 minutes at 4°C. Protein abundance was determined by BCA protein assay (Thermo Fisher Scientific). Protein was denatured by adding NuPAGE LDS buffer and dithiothreitol reducing buffer (Invitrogen) and adding samples to a heatblock for 95°C for 5 minutes. Equal amounts of denatured protein, generally 30 μg per sample, were loaded in NuPAGE precast gels (Invitrogen), fractioned by SDS-PAGE and electrotransferred to nitrocellulose membranes using the iBLOT2 system (Invitrogen).

Nonspecific block of membranes used 5% nonfat milk solution for 30 minutes at room temperature (RT) and were probed with the corresponding primary antibody at generally 1:1,000 dilution overnight at 4°C. Subsequently, the primary antibody was washed away using TBST, and a secondary antibody incubation with the corresponding horseradish peroxidase (HRP)-conjugated antibody at 1:5,000 dilution was carried out for 1 hour at RT. Antibodies used in these analyses are listed in Supplementary Table S3.

### RT-qPCR

RNA from cells was extracted using RNeasy Plus Kit (Qiagen) as per the manufacturer’s instructions, which included a DNase digestion step. Eluted RNA was quantified with a NanoDrop spectrophotometer (Thermo Fisher Scientific). Equal amounts of RNA were reverse transcribed and amplified using either TaqMan RNA-to-CT 1-Step Kit (Applied Biosystems) or High-Capacity cDNA Reverse Transcription Kit (Applied Biosystems) followed by TaqMan Universal PCR Master Max (Applied Biosystems). qPCR reactions occurred using the ViiA 7 Real-Time PCR System (Applied Biosystems). The ΔΔCt values per gene were calculated by relating each individual Ct value to its *GAPDH* housekeeping control and then normalized to the individual or averaged control condition. The relative quantification was calculated as 2^−ΔΔCt^ and presented as Gene Expression. TaqMan gene expression assay probes used in these studies are listed in Supplementary Table S3.

### Cell-cycle determination

Colo201, CCK81 shATF6, and shCTNNB1 cells were plated at 3e5 cell/well of a six-well dish and treated with 0.5 μg/mL of Dox for 72 hours. For 5-ethynyl-2′-deoxyuridine (EdU) incorporation studies, cells were then pulsed with 50 μmol/L EdU (Click-iT EdU Flow Cytometry, Invitrogen) culture media for 1 hour. Cells were then dissociated, counted, and collected for fixation/permeabilization before proceeding with the Click-it EdU reaction. Finally, DNA content was stained in saponin perm/fix solution using Hoechst Ready Flow Regeant (Thermo Fisher Scientific) as per the manufacturer’s instructions. The samples were then analyzed using a FACSymphony analyzer (BD) and, using FLowJo (RRID: SCR_008520), single cells were allocated into cell-cycle phases according to their EdU positivity and DNA content (EdU+ : S-phase; EdU-/DNA = 2n: G0/G1; EdU-/DNA = 4n: G2/M).

For propidium iodide (PI) incorporation studies, 1 × 10^6^ cells were fixed in ice-cold 70% ethanol overnight at <4°C. After washing in PBS, samples were treated with 100 μg/mL RNase (Zymo Research) for 15 minutes at RT, followed by incubation with 50 μg/mL PI for 20 minutes at RT. Samples were analyzed using a FACSymphony analyzer (BD), and data were collected in linear scale for better DNA content determination. Univariate modeling (either Watson pragmatic or Dean-Jett Fox) was used to create a fit to cell-cycle data based on statistics in the DNA content dimension and was performed with the FlowJo 10.10.0 software “cell cycle” function, constraining the model to equal G2 and G1 coefficients of variation (CVs).

### Measurement of seeding capacity

Using either a flat bottom 96-well plate (3595, Corning) or an ULA 96-well plate (7007, Corning), specified cell line was seeded at upper-left most well (A1) at an initial density of 2 × 10^5^ cell/well. Cell suspension was serially diluted 1:2 vertically downward (A1→A8). All cell-bearing wells in the column (A) were horizontally serially diluted 1:2 (A→I). All wells were then evenly loaded to be at 50 μL and then treated with or without Dox (1 μg/mL) at 50 μL to create a 100 μL culture at 0.5 μg/mL Dox treatment. For Ceapin studies, a 20 μmol/L concentration was loaded to create a 10 μmol/L treatment. Culture was then visualized via Incucyte and grown for approximately 7 days. Cell viability was determined using CellTiterGlo and plotted as a function of density versus raw luminescent units. To compare high-density versus low-density wells, viability was normalized to the no Dox control well of the same density. Cells were visualized with supplementation of Nuclight (Essen Bioscience) following manufacturer’s specifications and visualized using IncuCyte instrument (Sartorius).

### TOP.FLASH reporter activity

Measure of β-catenin transcriptional activity through the TCF optimal promoter (TOP.FLASH) assay to assess Wnt signaling activity is well described (42). CCK81 shATF6 and Colo201 shATF6.1 were stably transfected for TOP.FLASH using a piggyBac transposase system with a positive Cyan fluorescent protein. Cyan fluorescent protein^+^ cells were sorted and cultured. ATF6 knockdown occurred at a specified length of time with Dox (0.5 μg/mL), and firefly luminescence was detected using the Dual-Luciferase Reporter Assay System (Promega).

### RNA-seq

Colo201 shNTC, shATF6.1 and shATF6.2 and CCK81 shNTC, shATF6 were harvested in biologic triplicates at 0, 2, and 4 days after treatment with Dox (0.5 μg/mL). For bulk RNA-seq, RNA from 1 × 10^6^ cell was first extracted with RNeasy Plus Kit (Qiagen) as per the manufacturer’s protocol. Total RNA was quantified using Qubit RNA HS Assay Kit (Thermo Fisher Scientific), and quality was assessed using RNA ScreenTape on 4200 TapeStation (Agilent Technologies).

### Bulk RNA-seq

RNA-seq libraries were prepared using TruSeq RNA Sample Preparation Kit (Illumina). The libraries were sequenced on Illumina HiSeq 2500 sequencers to obtain on average 44 million 50-bp, stranded, single-end reads per sample. Raw sequencing outputs for all samples were mapped to the University of California, Santa Cruz (UCSC) human genome (GRCh38/hg38) using GSnap software (RRID: SCR_005483; ref. 39), allowing a maximum of two mismatches per 50-base sequence (parameters: -M 2 -n 10 -B 2 -i 1 -N 1 -w 200000 -E 1–pairmax-rna = 200000–clip-overlap). Gene expression counts were obtained by quantifying the number of reads uniquely mapping to each gene exonic locus. Lowly expressed genes were removed from all samples using a high-pass filter for genes with at least 15 counts in at least 10% of samples (4 of 33). Read counts were scaled by library size and quantile normalized, and precision weights were calculated using the voom function of the limma R package (RRID: SCR_010943; ref. 43) to yield log_2_ counts per million values of sufficiently covered genes. Differential gene expression analysis was performed with edgeR using quality weighting and robust empirical Bayes shrinkage (44). shATF6 − Dox (0 day) and shNTC + Dox served as controls for shATF6 + Dox samples (2, 4 days). Significantly downregulated or upregulated genes were defined by a log_2_ fold change absolute value >1 and a FDR <0.05. Gene set enrichment analyses were performed using fgsea (bioRxiv 2021.060012; parameters: stats = ranks, minSize = 5, maxSize = 500, and nperm = 1,000,000) on gene sets retrieved from the Molecular Signatures Database (complete HALLMARKS, C2 and C5 collections; refs. 45, 46).

### Single-nucleus RNA-seq

For single-nuclei RNA-seq PDM-272 organoid studies, organoids were treated in biologic triplicate with DMSO or Ceapin-A7 (3 μmol/L) for 48 hours and processed following the Nuclei Isolation for Single-Cell Multiome ATAC + Gene Expression Sequence (Demonstrated Protocol CG000365, Rev C, 10× Genomics) protocol. Briefly, treated PMD-272 organoid plugs were placed on ice to allow for plug softening and then resuspended in ice-cold PBS, pelleted, and dissociated using Accutase and gently pipetted to dissociate. Quality of dissociation, viability, and cell amount were determined using Vi-CELL BLU instrument (Beckman Coulter). Dissociated cells were then lysed, and nuclei were isolated and determined for quality isolation. The single-nucleus RNA-seq libraries was generated using Chromium Single-Cell Next GEM Reagent Kit (10× Genomics) and sequenced in HiSeq6000 (Illumina) as per the manufacturer’s instructions.

### Preprocessing and quality control

Single-nucleus RNA-seq data were processed using the Cell Ranger pipeline v3.0.2 (47) with default parameters. Quality control metrics were calculated for each cell using the “SingleCellExperiment” package (48). A threshold of 10% was ensured for the percentage of reads mapped to mitochondrial genes, and a minimum percentage of ribosomal RNA reads were applied to filter out low-quality cells. Only cells passing these criteria were included for downstream analysis.

### Normalization and feature selection

Cell count data were normalized by a size factor based on the total counts for each cell and then log-transformed using the “scran” package (49) to control for sequencing depth and RNA composition bias, along with variance stabilization. The “modelGeneVar” function from the “scran” package, with the trend method set to “loess”, identified highly variable genes for downstream analysis.

### Dimensionality reduction and clustering

Principal component analysis was performed on the matrix of highly variable genes using the “runPCA” function from the “Scater” package (50), applying centering and scaling to rows (genes). The top principal components explaining a substantial variance were used as inputs to the “runUMAP” function from the “scater” package to generate a two-dimensional Uniform Manifold Approximation and Projection (UMAP) representation. The “igraph::cluster_walktrap” function from the “igraph” package (51) applied UMAP coordinates to cluster cells based on their transcriptomic similarity.

### Scoring of cell states and visualization

The Wnt pathway gene set was derived from the study “Human colon organoids reveal distinct physiologic and oncogenic Wnt responses” by Michels and colleagues (52). Gene sets related to ER stress, UPR, apoptosis, stemness, and hallmarks were sourced from the Molecular Signatures Database (ref. 45). Furthermore, markers for mitosis and colon cell types were obtained from Seurat (53) and Haber and colleagues (54). These gene sets were scored at the cell level utilizing the “ScoreSignatures UCell” function from the “UCell” package (55).

Visualization of the distribution of scores and expression levels of gene sets across different cell clusters on the UMAP plots were generated using the “ggplot2” (56) and “dittoSeq” (57) packages.

## Proteomics

### Proteomics sample preparation

Organoid PDM-272 cells were harvested and lysed in HEPES buffer (20 mmol/L, pH 8.0) containing 9 mol/L urea and phosphatase inhibitors (1 mmol/L sodium orthovanadate, 2.5 mmol/l sodium pyrophosphate, and 1.0 mmol/L β-glycerophosphate). Lysates from three conditions were generated for global proteome analysis. The treatment conditions were DMSO, Ceapin-A7, and its inactive analog for 72 hours. Each condition was performed in four biological replicates for a total of 12 samples. Lysates were sonicated followed by centrifugation at 20,000 *g* for 20 minutes at 15°C. Protein concentration was measured using Bradford assay (Bio-Rad). Proteins (200 µg/condition) were reduced with 5 mmol/L DTT at 37°C for 1 hour followed by alkylation with 15 mmol/L iodoacetamide at RT for 20 minutes in the dark. Samples were diluted to a final concentration of 2 mol/L urea prior to digestion with Lys-C (Wako) at an enzyme: substrate ratio (E:S) of 1:50 at 37°C for 2 hours followed by trypsin (Promega) digestion at an E:S ratio of 1:50 at 37°C overnight. The digest mixtures were acidified with 20% trifluoroacetic acid (TFA) prior to solid-phase extraction using SOLA HRP SPE cartridge (10 mg absorbent; Thermo Fisher Scientific). Peptides were eluted with 2 × 0.25 mL of 60% acetonitrile (ACN)/0.1% TFA followed by peptide concentration measurement using a quantitative colorimetric peptide assay kit (Thermo Fisher Scientific). Equal amounts from each condition (100 μg) were subjected to lyophilization overnight.

### TMT labeling and global proteome analysis

The dried peptide mixtures were reconstituted in 100 μL of HEPES (100 mmol/L, pH 8.5). TMTPro reagent, channels 126 to 132N (20 μL at 500 μg/50 μL in ACN), was added to each of the 12 samples. Labeling was performed at RT for 1 hour. A small portion (2 μL) from each condition was mixed, desalted, and analyzed to determine labeling efficiency. The reaction was quenched with 5 μL of 5% hydroxylamine once labeling efficiency was determined to be at least 95%. Samples were mixed followed by acidification using 20% TFA and lyophilized overnight. The 12-plex TMT-labeled peptide mixture was desalted using a C_18_ cartridge (50 mg absorbent; Waters). Peptides were eluted with 3 × 0.25 mL of 60% ACN/0.1% TFA. Sample was lyophilized overnight and subjected to high-pH reverse phase fractionation on the Agilent 1100 high-performance liquid chromatography system (Agilent Technologies). The peptide mixture was reconstituted in 75 μL of solvent A (5% ACN/50 mmol/L ammonium bicarbonate, pH 8.0) and separated on a Zorbax 300Extend-C_18_, 3.5 μm, 4.6 × 150 mm column (Agilent Technologies) at a flow rate of 0.5 mL/minutes. A gradient from 15% to 45% solvent B (90% ACN/50 mmol/L ammonium bicarbonate, pH 8.0) was applied over 49 minutes with a total run time of 75 minutes. Ninety-six fractions were collected at 0.63 minutes interval, and every 25th fraction was combined into a set of 24 fractions. Fractions were acidified with 20% TFA and dried and desalted using SDB tips (GL Sciences) prior to mass spectrometry (MS) analysis.

### MS analysis

Desalted peptides were reconstituted in 2% ACN/0.1% formic acid/water and loaded onto Aurora Series 25 cm × 75 μm I.D. Column (IonOpticks) using the Dionex Ultimate 3000 RSLCnano ProFlow system (Thermo Fisher Scientific). Peptide separation was performed at 300 nL/minutes with a two-step linear gradient in which solvent B (0.1% formic acid/2% water/ACN) was increased from 4% to 30% over 68 minutes and then from 30% to 75% B over 4.9 minutes, with a total analysis time of 95 minutes. Peptides were analyzed using an Orbitrap Eclipse instrument (Thermo Fisher Scientific). For global proteome analysis, a real-time search against a human database was performed using an in-house instrument API program called InSeqAPI (58–60). Protein closeout was used (three distinct peptides/protein/run). Proteins from the Wnt signaling pathway were placed on the InSeqAPI inclusion list such that they were not subject to protein closeout. Precursor ions (MS1) were analyzed in the Orbitrap (250% normalized AGC target, 120,000 mass resolution, 50 ms maximum injection time), with the 10 most abundant species selected for MS2 fragmentation. Each precursor was isolated at a mass width of 0.5 Th followed by fragmentation using collision-induced dissociation [at 30 Normalized Collison Energy (NCE)], 150% normalized AGC target with a maximum injection time of 100 ms. Multiple fragment ions were isolated using synchronous precursor selection prior to MS3 HCD fragmentation (45 NCE, synchronous precursor selection notches = 8, AGC target = 3.0E5, and maximum injection time = 400 ms). MS3 scans were analyzed in the Orbitrap at 50,000× resolution.

### Bioinformatics

MS/MS data were searched using the Comet search algorithm against a concatenated forward–reverse target–decoy database (UniProtKBconcat, downloaded October 2023) consisting of *Homo sapiens* proteins and common contaminant sequences. Spectra were assigned using a precursor mass tolerance of 20 ppm and fragment ion settings for low-resolution MS/MS with a fragment ion bin tolerance and bin offset of 1.0005 and 0.4, respectively. Static modifications included carbamidomethyl cysteine (+57.0215 Da) and TMT tag (+304.2071 Da) on both the N-termini of the peptides and lysine residues. Variable modifications included oxidized methionine (+15.994 Da) and TMT tag on tyrosine residues (+304.2071 Da). Trypsin specificity with up to one miscleavage was specified. Peptide spectral matches were filtered at a 1% FDR. The TMT reporter ion quantification was performed using an in-house Mojave module (61) by calculating the highest peak within 20 ppm of theoretical reporter mass windows and correcting for isotope purities. This method determined the intensity of a protein per channel, as well as the sum of protein intensities across the studied proteome within samples, providing the relative abundance of a peptide across conditions. This value is referred to as “relative signal intensity” in text.

### Statistical analysis of MS data

The R package MSstatsTMT v.2.0.1 was used to preprocess PSM-level quantification before statistical analysis, to have protein quantification and to perform differential abundance analysis (62). Briefly, the preprocess filtered out TMT peaks in MS3 scans whose sum was less than 30,000 across all 12 channels. Peptides shorter than seven residues were also filtered out. The abundance of each identified protein was estimated by using a model fitted with Tukey median polish summarization, with imputation of missing values below a censoring threshold of 2^8^. MSstatsTMT estimated log_2_(fold change) and the standard error by the linear mixed-effects model for each protein. The inference procedure was adjusted by applying an empirical Bayes shrinkage. To test two-sided null hypothesis of no changes in abundance, the model-based test statistics were compared with the Student *t* test distribution with the degrees of freedom appropriate for each protein and each dataset. The resulting *P* values were adjusted to control the FDR with the Benjamini–Hochberg method. Based on principal component analysis, replicate 4 of inactive Ceapin-A7 treatment did not cluster with the rest of the replicates. Therefore, this sample was not included for statistical analysis.

### Mutational status determination of colorectal cancer organoids

Public sequencing data from the NCI Genomic Data Commons (https://portal.gdc.cancer.gov/cases/30ad857e-34d5-4ae1-8ca1-e962d10d6440?bioId=a14a4f36-9be1-4a6b-83a0-b9e00dcb4115) were used to make mutation calls for all colorectal cancer organoids (PDM-264, PDM-5, PDM-6, PDM-96, PDM-185, and PDM-272). Annotated are established driver mutations in colorectal cancer (Supplementary Table S4). For the RNF43 R132* stop-gain mutation in PDM-272, 147 of 147 reads showed the alteration, indicating that it is homozygous.

### Statistics

All values are represented as the arithmetic mean ± SEM if not otherwise indicated in the figure legends. All statistical analyses were performed using GraphPad Prism 10 (GraphPad Software, Inc.; RRID: SCR_002798). Unpaired two-tailed Student's t-tests were used to assess differences between the mean +/- SEM of two groups unless otherwise stated and significance is as follows: *, *P* < 0.05; **, *P* < 0.005; ***, *P* < 0.001; and ****, *P* < 0.0001. A *P* value > 0.05 was considered nonsignificant (ns).

### Data availability

The RNA-seq analyses of Colo201 and CCK81 cells used in this study have been deposited in the Gene Expression Omnibus with accession code GSE273988. The single-nuclei RNA-seq analysis is also deposited in the Gene Expression Omnibus with accession code GSE274172. Organoid proteomic work, including raw data files and associated meta files as well as quantitation data, have been deposited and can be accessed on MassIVE (MassIVE ID: MSV000095581). All other raw data are available upon request from the corresponding author.

## Results

### Several solid malignancies display elevated expression of ATF6-induced genes

**Figure 1:**
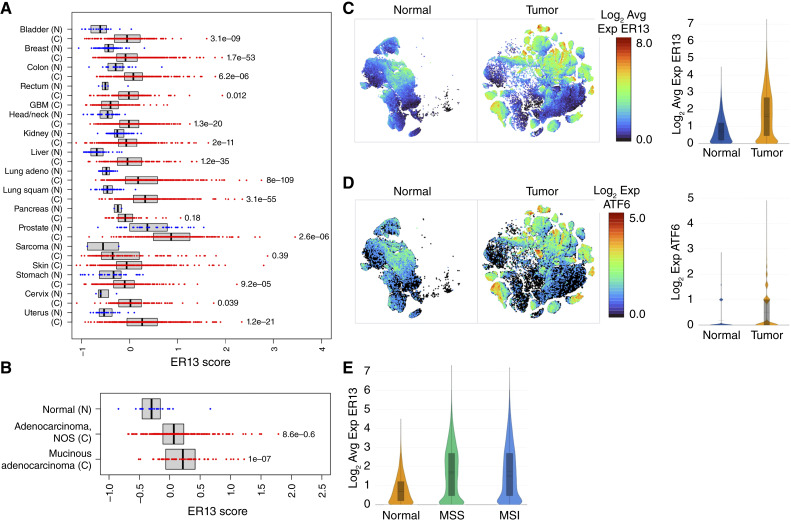
Several solid malignancies display elevated mRNA expression of ATF6 target genes. **A,** Expression sum of 13 known ATF6 target genes (ER13 score) based on tissue specimen data from TCGA. ER13 score comparison made respective to each tissue type between cancerous relative to normal tissue. **B,** ER13 score of patient samples from colorectal cancer subsets of mucinous or not-otherwise-stated adenocarcinoma from malignant and normal samples in TCGA. **C,** Average log_2_ expression ER13 single-cell expression of colonic epithelium cells from normal or tumor tissue from Pelka and colleagues (40). **D,***ATF6* expression of colonic epithelium cells from normal or tumor tissue. **E,** Average ER13 single-cell expression of colonic epithelium cells from normal or tumor tissue stratified by microsatellite stable or instable tumor cells. Avg, average; C, cancerous; Exp, expression; GBM, glioblastoma; MSI, microsatellite instable; MSS, microsatellite stable; N, normal; NOS, not otherwise stated.

To explore whether specific cancers may co-opt ATF6, we interrogated TCGA databases for the mRNA expression of a set of 13 genes (ER13) that were previously established to be induced by ectopic expression of nATF6 (Supplementary Table S1; ref. 36). We calculated an “ER13 score” based on the sum of expression of these genes across various tumor tissues, as earlier performed for IRE1α (28). Several major solid-tissue malignancies, including breast, colon, liver, lung, and uterine cancers, displayed significantly elevated ER13 scores in tumors as compared with normal tissue specimens; whereas several other malignancies showed no change, including cancers of the cervix, skin, and pancreas (Fig. 1A). We elected to focus on colorectal cancer, given its high prevalence, poor clinical prognosis in late-stage disease, and unmet need for novel therapies (63). Although colonic mucin production requires ER facilitation (64), both colorectal cancer mucinous adenocarcinoma and adenocarcinoma not otherwise specified showed markedly higher ER13 scores than normal tissue (Fig. 1B), suggesting that increased mucin production is not a major driver of ATF6 activity in colorectal cancer. As TCGA results rely on bulk RNA-seq data and thus may be confounded by the high cellular heterogeneity of colorectal cancer (65), we leveraged a recently reported scRNA-seq patient dataset, obtained from 60 colon tumors and 36 adjacent normal colon tissue samples (40). Analysis of these data revealed that tumor cells displayed increased ER13 expression (Fig. 1C) as well as *ATF6* expression, as compared with colonic normal cells (Fig. 1D). Each of the 13 genes had a varying elevated expression in tumor versus normal cell compartments (Supplementary Fig. S1A) and spatially corresponded with increased ER13 and *ATF6* levels (Supplementary Fig. S1B). Notably, MS stability also did not correlate with altered ER13 scores (Fig. 1E), excluding this feature as a major driver of increased ATF6 activity. Taken together, these results reveal that malignant colonic tissues exhibit elevated mRNA expression and transcriptional activity of ATF6.

### ATF6 disruption impairs growth of multiple colorectal cancer cell lines

**Figure 2:**
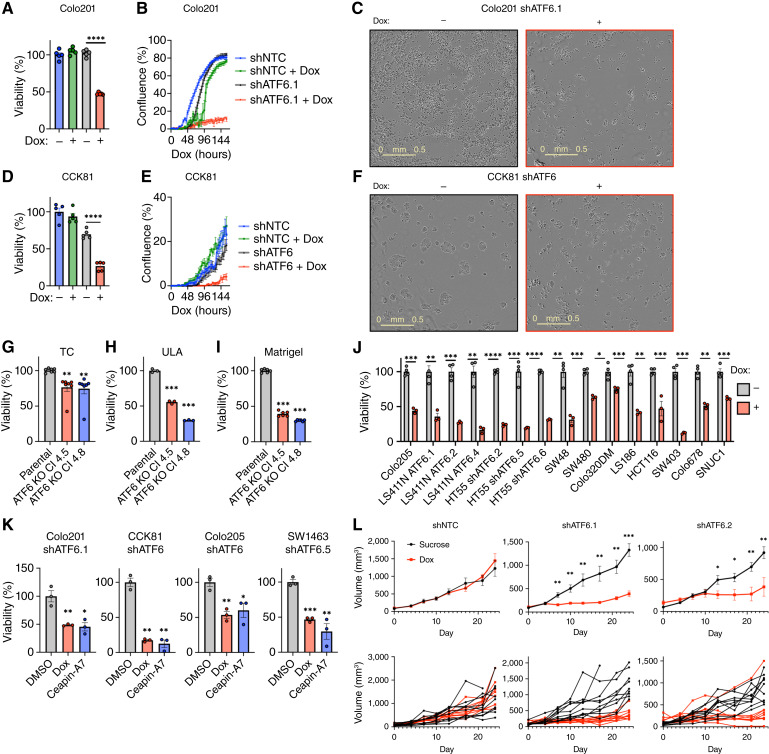
Disruption of ATF6 attenuates growth of multiple colorectal cancer cell lines *in vitro* and *in vivo*. **A,** Viability of Colo201 cells expressing shNTC or shATF6 grown in the presence or absence of Dox (0.5 μg/mL) for 7 days (*n* = 5). **B,** Kinetics of growth confluence of Colo201 cells expressing shNTC or shATF6 as treated in **A**. **C,** Representative light microscopy images of cells in **B** after 7-day treatment at 4× magnification. **D,** Viability of CCK81 cells expressing shNTC or shATF6 treated as in **A** (*n* = 5). **E,** Confluence of CCK81 cells expressing shNTC or shATF6 treated as in **A**. **F,** Representative light microscopy images of cells in **D** after 7-day treatment. **G–I,** Viability of parental (WT) Colo201 cells or clonal derivatives harboring CRISPR-based ATF6 KO [KO clones (Cl) 4.5 and 4.8] grown on standard TC plates (**G**), ULA plates (**H**), or in Matrigel (**I**; *n* ≥ 3). **J,** Viability of various colorectal cancer shATF6 cell lines after 7 days of growth in the absence or presence of Dox (*n* ≥ 3). **K,** Viability of selectively inducible shATF6 colorectal cancer cell lines after 7 days of growth in the presence of Dox or Ceapin-A7 (10 μmol/L; *n* = 3). **L,** Tumor growth kinetics of Colo201 cell lines expressing inducible shNTC, shATF6.1, or shATF6.2. Following s.c. injection and tumor establishment, tumor-bearing mice were offered water supplemented with 5% sucrose without or with Dox (0.5 mg/mL; *n* = 10 per group). WT, wild-type. *, *P* < 0.05; **. *P* < 0.005; ***, *P* < 0.001; ****, *P* < 0.0001.

To assess the importance of ATF6 activity for colorectal cancer growth, we disrupted *ATF6* expression in human colorectal cancer cell lines. We constructed a tetracycline-selectable plasmid encoding a set of three Dox-inducible shRNAs that perturb *ATF6* mRNA expression and stably transfected this plasmid into the human Colo201 and CCK81 colorectal cancer cell lines. For Colo201 cells, we established two sister clones (Colo201 shATF6.1 and shATF6.2), whereas for CCK81 cells, we established a pooled line (CCK81 shATF6). Dox treatment efficiently depleted the ATF6 protein within 2 to 4 days in cells expressing the shATF6 plasmid as compared with a corresponding NTC shRNA construct (shNTC; Supplementary Fig. S2A). Strikingly, ATF6 knockdown significantly reduced viability and attenuated proliferation in both cell lines (Fig. 2A–F).

Earlier studies from our laboratory demonstrated that dependency on IRE1α in triple-negative breast cancer cell lines emerges upon cell propagation in 3D settings but not under standard TC conditions (29). In contrast, we observed a clear ATF6 dependency in Colo201 and CCK81 cells grown either as monolayers on standard TC plates or in 3D on ULA plates (Supplementary Fig. S2B and S2C). To corroborate the requirement for ATF6 through a different strategy, we disrupted the *ATF6* gene in Colo201 cells via CRISPR/Cas9, which led to a complete and constitutive loss of ATF6 in two independent CRISPR clones (4.5, 4.8; Supplementary Fig. S2D). In contrast to the *ATF6* knockdown clones, the *ATF6* KO clones displayed only a modest reduction in viability as compared with parental cells under standard TC conditions (Fig. 2G); nevertheless, upon culture on ULA plates or in Matrigel, they showed a stronger reduction in viability (Fig. 2H and I). The reduced impact of constitutive *ATF6* KO versus inducible knockdown could be due to a compensatory adaptation that enables rare cells to survive despite the stable loss of *ATF6*. To circumvent this caveat, we leveraged ASOs as an alternative conditional silencing approach. We packaged two different ASOs targeting *ATF6* or a negative control ASO targeting *α-Synuclein* (*SNCA*) into lipid nanoparticles and added these to Colo201 cells. The *ATF6* ASOs induced a substantial depletion of ATF6 protein within 2 days, whereas the *SNCA* ASO did not (Supplementary Fig. S2E). ASO-mediated ATF6 disruption decreased Colo201 cell viability comparably with Dox-induced ATF6 knockdown, whereas the *SNCA* ASO had no effect (Supplementary Fig. S2F), further confirming the specific ATF6 requirement in this colorectal cancer cell line.

To examine the scope of ATF6 dependency in colorectal cancer models, we transfected a cohort of 12 additional human colorectal cancer lines with our inducible ATF6 shRNA construct (Supplementary Fig. S2G and S2H). Remarkably, all but the RKO cell line (Supplementary Fig. S2I and S2J) showed significant reductions in viability upon ATF6 silencing (Fig. 2J), indicating a broad ATF6 dependency in diverse colorectal cancer backgrounds. To verify the functional requirement for ATF6, we leveraged a previously established small-molecule inhibitor of ATF6 activation, Ceapin-A7 (66). This compound tethers ER-resident ATF6 to the peroxisome-resident transmembrane transporter, ABCD3 (66), which prevents ATF6 from translocating to the Golgi compartment and thereby blocks its processing into active nATF6 (67, 68). As expected, Ceapin-A7 inhibited nATF6 generation upon treatment of Colo201 cells with the potent pharmacologic ER stress inducer Tg (Supplementary Fig. S2K). Importantly, Ceapin-A7 impaired viability of several cell lines that were sensitive to ATF6 knockdown (Fig. 2K; Supplementary Fig. S2L and S2M) while having little effect on the RKO line (Supplementary Fig. S2N), in keeping with the shRNA results. Finally, in contrast to ATF6 knockdown, silencing of another branch of the UPR, IRE1, did not impact the viability of Colo201 cells (Supplementary Fig. S2O and S2P), illustrating their distinct dependency on ATF6.

To investigate the importance of ATF6 for colorectal cancer growth *in vivo*, we first validated the effectiveness of our Dox-inducible ATF6 shRNA approach in Colo201 tumor xenografts grown subcutaneously in mice. Reassuringly, supplementation of the drinking water with sucrose plus Dox led to efficient depletion of the ATF6 protein in tumors expressing the shATF6 but not the shNTC plasmid (Supplementary Fig. S2Q). To assess the impact of ATF6 knockdown on tumor progression, we treated mice harboring pre-established palpable tumors with sucrose-supplemented water lacking or containing Dox and tracked tumor volumes over 3 weeks. Although Dox supplementation did not affect growth of shNTC tumors, it significantly attenuated tumor progression of each of the shATF6 clones by approximately 70% (Fig. 2L). In keeping with these results, the two independent Colo201 clones harboring CRISPR-based ATF6 KO grew in xenografted mice at reduced rates as compared with parental Colo201 cells (Supplementary Fig. S2R). Furthermore, induction of ATF6 knockdown in pre-established CCK81 s.c. xenografts also substantially inhibited tumor progression (Supplementary Fig. S2S). Together, these results reveal a previously unrecognized *intrinsic *dependency in several colorectal cancer cell lines on ATF6, not only for *in vitro* growth but also for *in vivo* tumor progression, distinctly from earlier reports showing ATF6 requirement during experimentally induced stress (69–71).

### ATF6 disruption in colorectal cancer lines induces cell-cycle arrest

Given known coordination between UPR branches (72), we examined whether ATF6 silencing affected IRE1 and PERK signaling. In Colo201 cells, ATF6 knockdown increased the IRE1 mediator XBP1s and the PERK conduits ATF4 and CHOP, but the induction was modest as compared with the robust upregulation of these factors in response to Tg-driven ER stress (Supplementary Fig. S3A). Moreover, in CCK81 cells, ATF6 silencing did not affect XBP1s, ATF4, or CHOP levels (Supplementary Fig. S3A). These results suggest that ATF6 disruption attenuates Colo201 and CCK81 growth without provoking a major coordinated engagement of IRE1 and PERK.

**Figure 3:**
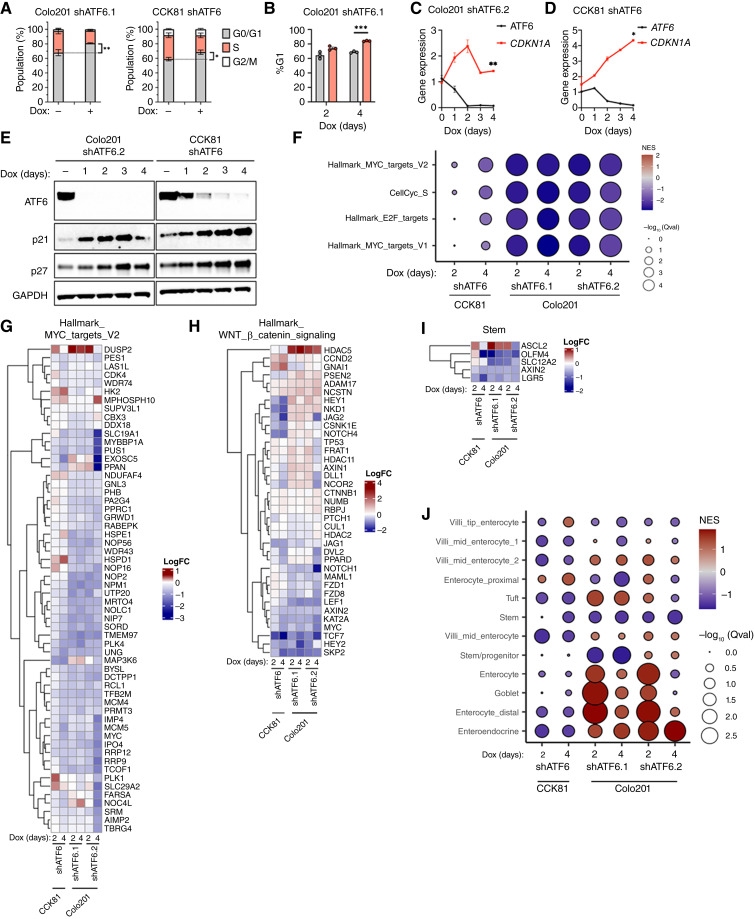
ATF6 silencing attenuates cell-cycle progression and downregulates cell-cycle drivers. **A,** Colo201 and CCK81 cells expressing inducible shATF6 were treated with or without Dox for 3 days and subjected to cell-cycle analysis by EdU incorporation (*n* = 3). **B,** G1 phase abundance of Colo201 shATF6.1 cells after treatment with or without Dox for specified treatment lengths as determined by cell-cycle analysis by PI staining (*n* = 3). **C,** Colo201 cells expressing inducible shATF6 were treated with Dox for 0 to 4 days and mRNA expression of *ATF6* and *CDKN1A*/p21 was determined by qPCR (*n* = 2 per group). **D,** CCK81 cells expressing inducible shATF6 were treated and similarly analyzed as in **B**. **E,** Colo201 and CCK81 cells expressing inducible shATF6 were treated without (−) or with Dox for 0 to 4 days, and protein levels of ATF6, p21(*CDKN1A*), and p27 were determined by immunoblotting. Cells treated without Dox harvested on day 4. **F,** GSEA plots of hallmark gene sets. **G,** Heatmap of hallmark MYC targets v2. **H,** Heatmap of hallmark Wnt/β-catenin signaling. **I,** Heatmap of stem gene set. **J,** GSEA plots of stem and specific differentiated cell type gene sets. CellCyc, cell cycle; NES, Normalized Enrichment Score. ; *, *P* < 0.05; **, *P* < 0.005; ***, *P* < 0.001.

Because the UPR plays dynamic roles in apoptosis control (73, 74), we next interrogated whether apoptotic caspase activation could account for the reduction in cell viability upon ATF6 silencing. ATF6 knockdown in CCK81 cells did increase caspase-3/7 activity, which could be blocked by addition of the pan-caspase inhibitor zVAD (Supplementary Fig. S3B). Nevertheless, zVAD addition did not restore viability of ATF6-silenced Colo201 or CCK81 cells (Supplementary Fig. S3C), excluding apoptotic caspase activation from being an essential driver of the observed growth disruption. We reasoned alternatively that ATF6 silencing could impair growth by blocking cell-cycle progression. To investigate this possibility, we performed flow cytometric analysis of EdU incorporation into DNA as a measure of cell-cycle progression. ATF6 silencing in Colo201 and CCK81 cells caused their accumulation within the G1 phase of the cell cycle (Fig. 3A). Further kinetic analysis revealed that the inhibition of cell-cycle progression occurred within the first 2 days of Dox addition and extended throughout a 4-day treatment (Fig. 3B).

Consistent with the latter results, ATF6 knockdown increased mRNA expression of the cyclin-dependent kinase inhibitor (CDKi) p21/*CDKN1A* within the first 2 days of Dox addition (Fig. 3C and D), as well as protein levels of both p21 and the post-transcriptionally regulated CDKi p27 (Fig. 3E). ATF6 knockdown also induced accumulation of p21 and p27 in several other ATF6-dependent colorectal cancer cell lines (Supplementary Fig. S3D). Moreover, consistent with the *in vitro* findings, ATF6 depletion in Colo201 tumors *in vivo* increased p21 levels as compared with control (Supplementary Fig. S3E). Taken together, these results establish a significant ATF6 requirement for cell-cycle progression in these colorectal cancer lines.

### ATF6 silencing attenuates genes involved in cell-cycle control and in Myc and Wnt signaling

To gain further insights into how ATF6 supports cell-cycle progression, we performed a comparative bulk RNA-seq study of Colo201 and CCK81 cells after the induction of shATF6 versus shNTC (Supplementary Fig. S3F). As expected, Dox addition decreased mRNA abundance of *ATF6* as well as several ATF6 target genes in cells expressing shATF6 but not shNTC (Supplementary Fig. S3G and S3H). Further confirming on-target shATF6 activity, Dox treatment of cells expressing ATF6 shRNA as compared with shNTC did not decrease the mRNA levels of *MAP2K3*, a transcript predicted by sequence comparison to be one of very few potential off-targets of the ATF6 shRNAs we used (Supplementary Fig. S3I; Supplementary Table S2). In keeping with the data above, gene-set enrichment analysis of the RNA-seq results revealed significant downregulation upon ATF6 knockdown of several cell-cycle gene sets, including cell-cycle S-phase, and E2F target hallmarks (Fig. 3F; Supplementary Fig S3J). The Wnt/β-catenin pathway—a pivotal oncogenic driver in colorectal cancer (75)—promotes aberrant proliferation by inducing *Myc* expression (76). Strikingly, ATF6 silencing attenuated both the Myc signaling and Wnt/β-catenin signaling gene sets (Fig. 3G and H; Supplementary Fig S3J). Given the critical role of Wnt signaling in driving stemlike function in colorectal cancer cells, we prioritized examining the impact of ATF6 silencing on gene sets associated with stemness and with several colonic tissue differentiation modules. ATF6 knockdown in both cell lines diminished expression of the stem gene set (Fig. 3I) and increased expression of specific differentiation lineages: enterocyte proximal for CCK81 and enterocyte/goblet for Colo201 (Fig. 3J). Of note, ATF6 silencing did not significantly impact expression of the hallmark UPR (Supplementary Fig. S3K), in agreement with the finding that ATF6 disruption did not provoke broad ER stress. Intriguingly, ATF6 depletion increased expression of gene sets linked to adipogenesis, apoptosis, coagulation, complement, and fatty acid metabolism (Supplementary Fig. S3L).

### ATF6 disruption decreases Wnt signaling and stemness

**Figure 4:**
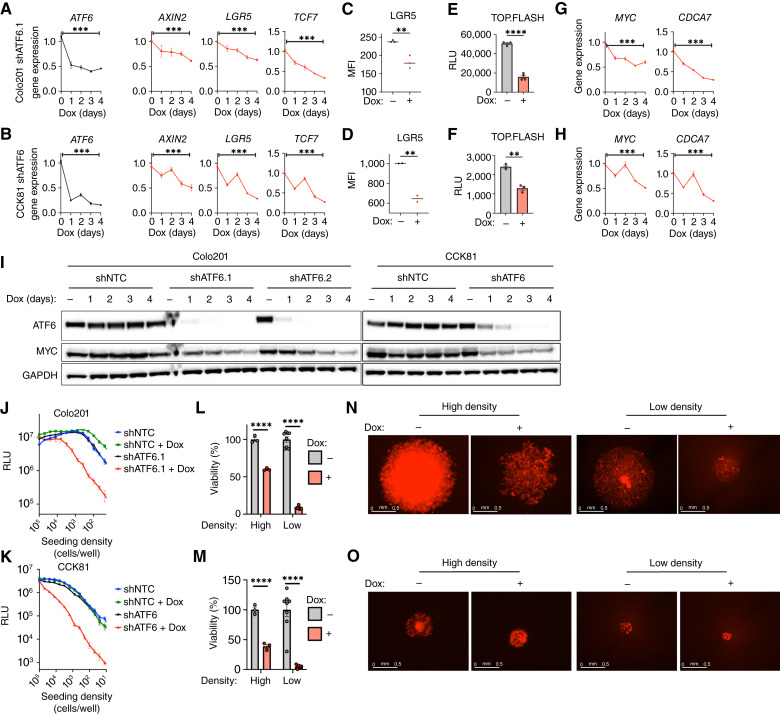
ATF6 disruption perturbs Myc and Wnt signaling and decreases colorectal cancer cell seeding capacity. **A,** Effect of ATF6 knockdown on Wnt signaling genes. Colo201 shATF6.1 cells were incubated for the indicated time with Dox (0.5 μg/mL) and analyzed by RT-qPCR for expression of *ATF6* and Wnt signaling genes (*AXIN2*, *LGR5*, and *TCF7*) mRNA (*n* = 3). **B,** Gene expression of CCK81 shATF6 cells as treated and analyzed in **A** (*n* = 3). **C,** Effect of ATF6 knockdown on LGR5 surface expression. Colo201 shATF6.1 cells were treated with or without Dox (0.5 μg/mL) for 3 days and analyzed by flow cytometry for LGR5 surface expression (*n* = 3). **D,** LGR5 expression of CCK81 shATF6 cells as in **C** (*n* = 2). **E,** Raw luminescence units of TOP.FLASH reporter Colo201 shATF6.1 cells after 2-day treatment of Dox (0.5 μg/mL) to report Wnt signaling activity (*n* = 4). **F,** TOP.FLASH expression of CCK81 shATF6 cells as in **E** (*n* = 3). **G,** Effect of ATF6 knockdown on Myc signaling genes. Colo201 shATF6.1 cells were incubated for the indicated time with Dox (0.5 μg/mL) and analyzed by RT-qPCR for expression of Myc signaling genes (*MYC*, *CDCA7*) mRNA (*n* = 3). **H,** Gene expression of CCK81 shATF6 cells as in **G** (*n* = 3). **I,** Effect of ATF6 knockdown on Myc expression. Colo201 shNTC, shATF6.1 and shATF6.2 cells and CCK81 shNTC, shATF6 cells were incubated for the indicated time with Dox (0.5 μg/mL) and analyzed by immunoblotting. Cells treated without (−) Dox were harvested on day 4. **J,** Effect of ATF6 knockdown on seeding potential. Colo201 shNTC and shATF6.2 cells were seeded at decreasing concentrations of 96-well and grown in the presence or absence of Dox (0.5 μg/mL) for 7-day. Cell abundance determined by raw luminescence units detected from CellTiterGlo assay (*n* ≥ 3). **K,** Seeding potential of CCK81 shNTC and CCK81 shATF6 cells as in **J** (*n* ≥ 3). **L,** Viability of Colo201 shATF6.2 cells seeded at high (1.25e4 cell/well) or low (2e2 cell/well) densities, as described in **J** (*n* ≥ 3). **M,** Viability of CCK81 shATF6 cells seeded at high (1.25e4 cell/well) or low (2e2 cell/well) densities, as described in **L** (*n* ≥ 3). **N,** Fluorescent microscopy images of Colo201 shATF6.1 cells seeded at high (1.25e4 cell/well) or low (4e2 cell/well) densities in ULA wells after 7 days growth in absence or presence of Dox (0.5 μg/mL) as visualized by nuclei signal (red). **O,** Fluorescent microscopy images of CCK81 shATF6 cells, as described in **N**. MFI, Median Fluorescence Intensity; RLU, raw luminescence unit. **, *P* < 0.005; ***, *P* < 0.001; ****, *P* < 0.0001.

Genetic alterations that hyperactivate the Wnt pathway play a central role in colorectal cancer (77–80). In a majority of colorectal cancer cases, biallelic mutations of *APC*—which encodes a key component of the β-catenin destruction complex—lead to unmitigated stabilization of β-catenin (81), thereby driving constitutive pathway activation independent of Wnt ligands. Colo201 cells harbor a truncating mutation in *APC* as well as an amino acid–substituting mutation in β-catenin (*CTNNB1*; *N287S*; ref. 82). CCK81 cells possess an inactivating mutational substitution in *APC* (Y159C) and a stabilizing mutation in *CTNNB1* (T41A; ref. 83). Inducible silencing of ATF6 coincided with transcriptional downregulation of several β-catenin target genes, namely, *AXIN2*, *LGR5* and *TCF7*, as early as 24 hours after Dox addition (Fig. 4A and B). Flow cytometric analysis further indicated that ATF6 knockdown also decreased the cell-surface expression of LGR5 (Fig. 4C and D; ref. 84). To assess Wnt pathway activity more directly, we transfected Colo201 shATF6.1 and CCK81 shATF6 cells with a previously established transcriptional reporter of Wnt signaling, dubbed TOP.FLASH (42). ATF6 depletion markedly reduced TOP.FLASH activity (Fig. 4E and F), confirming decreased Wnt/β-catenin signaling. Furthermore, ATF6 knockdown decreased mRNA expression of *MYC* and its transcriptional target *CDCA7* (Fig. 4G and H; ref. 85), with similar kinetics to the reduction in Wnt signaling targets (Fig. 4A and B). ATF6 silencing also decreased MYC protein levels, which remained low throughout 4 days of Dox treatment (Fig. 4I). In keeping with the shRNA data, *ATF6* targeting by ASOs also substantially reduced the expression of MYC in Colo201 cells (Supplementary Fig. S4A). ATF6 ASO treatment decreased ATF6 and MYC expression and reduced viability similiar to Dox-induced silencing also in HT29 cells (Supplementary Fig. S4B and S4C).

Wnt signaling in the intestinal crypt augments epithelial stemness, which in the context of cancer enables a miniscule number of transformed initiating cells to give rise autonomously to a malignant tumor (86–88). Because ATF6 silencing downregulated *LGR5*—an established transcriptional target of β-catenin and a classical indicator of colorectal cancer stemness (12)—as well as depleted the Stem gene set, we examined whether ATF6 disruption affects stemness at a functional level. To this end, we seeded Colo201 and CCK81 cells at progressively lower densities and assessed their viability after 7 days of culture in the absence or presence of Dox. Consistent with our earlier data, ATF6 silencing reduced the viability of cells seeded at high density by 50% to 60%; remarkably, ATF6 knockdown had an even stronger impact on cells seeded at low density, reducing viability by 95% relative to controls (Fig. 4J and K). Furthermore, ATF6 knockdown decreased the seeding capacity of both cell lines by at least 10-fold (Fig. 4L and M). Similar to ATF6 depletion, Ceapin-A7 treatment also reduced the seeding capacity of Colo201 cells (Supplementary Fig. S4D and S4E). ATF6 silencing also led to stronger viability losses at low versus high seeding density in several additional colorectal cancer lines (Supplementary Fig. S4F). We further examined the effect of ATF6 disruption on stemness by assessing the spheroid-forming capacity of cells cultured on ULA plates, which revealed a similar ATF6 dependency evident by decreased cell viability (Supplementary Fig. S4G) and altered spheroid morphology (Fig. 4N and O). These results indicate that ATF6 facilitates colorectal cancer stemness.

**Figure 5:**
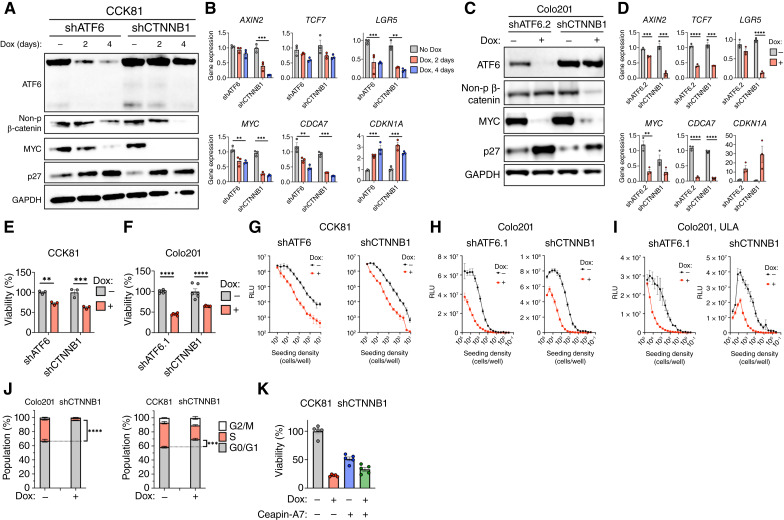
β-catenin knockdown phenocopies ATF6 silencing. **A,** Validation of knockdown in CCK81 cells. CCK81 shATF6 cells and CCK81 shCTNNB1 cells were incubated with Dox (0.5 μg/mL) for 2 and 4 days and analyzed by immunoblotting. Cells treated without Dox (−) were harvested after 4 days. **B,** CCK81 shATF6 cells and CCK81 shCTNNB1 cells were grown in the absence or presence of Dox for 2 and 4 days and were analyzed by RT-qPCR for Wnt signaling (*AXIN2*, *LGR5*, and *TCF7*), Myc signaling (*MYC* and *CDCA7*), and *CDKN1A* mRNA (*n* = 3). **C,** Validation of knockdown in Colo201 cells. Colo201 shATF6.2 cells and Colo201 shCTNNB1 cells were incubated with Dox (0.5 μg/mL) for 3 days and analyzed by immunoblotting. **D,** Colo201 shATF6.2 cells and Colo201 shCTNNB1 cells were grown in the presence of Dox for 3 days and were analyzed by RT-qPCR for Wnt signaling (*AXIN2*, *LGR5*, and *TCF7*), Myc signaling (*MYC* and *CDCA7*), and *CDKN1A* mRNA (*n* = 3). **E,** Viability of CCK81 shATF6 cells and CCK81 shCTNNB1 cells grown in the presence or absence of Dox (0.5 μg/mL) for 5 days (*n* = 3). **F,** Viability of Colo201 shATF6.1 cells and Colo201 shCTNNB1 cells grown in the presence or absence of Dox (0.5 μg/mL) for 7 days (*n* = 6). **G,** Quantification of cell abundance as determined by relative luminescence units of CCK81 shATF6 cells and CCK81 shCTNNB1 cells that were seeded at specified density and grown with or without Dox (0.5 μg/mL) for 7 days. **H,** Cell abundance of Colo201 shATF6.1 cells and Colo201 shCTNNB1 cells, as described in **G**. **I,** Cell abundance of Colo201 shATF6 cells and Colo201 shCTNNB1 cells grown in ULA plates, as described in **G**. **J,** Colo201 and CCK81 shCTNNB1 cells were treated with or without Dox for 3 days and subjected to cell-cycle analysis by EdU incorporation (*n* = 3). **K,** Viability of CCK81 shCTNNB1 cells treated separately with Dox (0.5 μg/mL), Ceapin-A7 (10 μmol/L), or concomitantly after 7 days (*n* = 5). RLU, relative luminescence unit. ** , *P* < 0.005; ***, *P* < 0.001; ****, *P* < 0.0001.

To compare ATF6 silencing with direct Wnt-pathway disruption, we used a parallel inducible silencing strategy for β-catenin (*CTNNB1*). Dox treatment of CCK81 cells expressing the inducible *CTNNB1* shRNA plasmid led to a nearly complete depletion of β-catenin without significantly affecting ATF6 levels (Fig. 5A). Knockdown of either ATF6 or β-catenin decreased MYC and increased p27 protein levels (Fig. 5A). Furthermore, silencing of either ATF6 or β-catenin decreased mRNA levels of *TCF7*, *LGR5*, *MYC*, and *CDCA7* and increased *CDKN1A* transcripts (Fig. 5B). We obtained similar results in Colo201 cells (Fig. 5C and D). Moreover, knockdown of either ATF6 or β-catenin comparably reduced cell viability (Fig. 5E and F), seeding potential (Fig. 5G and H), and spheroid formation capacity (Fig. 5I) in CCK81 and Colo201 cells. Akin to the effect of ATF6 knockdown on cell-cycle progression (Fig. 3A), β-catenin silencing also led to a G1 accumulation in both CCK81 and Colo201 cells (Fig. 5J). Of note, concomitant β-catenin knockdown and ATF6 inhibition attenuated CCK81 growth to a similar extent as each singular intervention (Fig. 5K), suggesting functional convergence between the two factors. Silencing of ATF6 or β-catenin also comparatively decreased the seeding capacity of HCT116 cells (Supplementary Fig. S5A and S5B). RKO—the only colorectal cancer line we examined that was not sensitive to ATF6 knockdown (Supplementary Fig. S2I and S2N)—differs from the ATF6-dependent lines in that it lacks dysregulating mutations in the Wnt pathway (89). Consistent with this difference in the mutational status, RKO cells showed lower levels of active β-catenin as compared with HCT116 cells; moreover, whereas ATF6 knockdown did not downregulate MYC or upregulate p27 in RKO cells, both ATF6 knockdown and β-catenin silencing altered the expression of these markers in HCT116 cells (Supplementary Fig. S5C). Furthermore, neither ATF6 knockdown nor β-catenin silencing inhibited RKO growth (Supplementary Fig. S5D). Together, these results indicate that the dependency of colorectal cancer lines on ATF6 may occur in the context of mutationally dysregulated Wnt signaling.

### ATF6 inhibition attenuates malignant intestinal organoid growth

**Figure 6:**
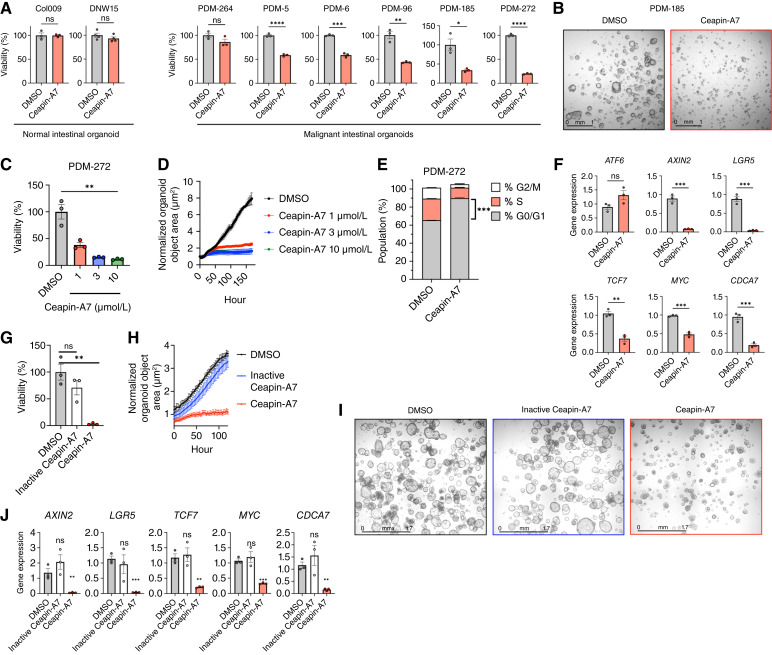
Selective ATF6 inhibition attenuates colorectal cancer organoid growth while inhibiting Wnt-pathway activity. **A,** Viability of normal (Col009 and DNW15) or malignant (PDM-264, PDM-5, PDM-6, PDM-96, PDM-185, and PDM-272) intestinal organoids treated with Ceapin-A7 (10 μmol/L) for 7 days (*n* = 3). **B,** Brightfield 4× visualization of PDM-185 growth in the absence (DMSO) or presence of Ceapin-A7 (10 μmol/L) for 7 days. **C,** Viability of PDM-272 organoid after 7 days of Ceapin-A7 treatment at the specified concentration (*n* = 3). **D,** Organoid confluence of PDM-272, as described in **C,** as quantified by Incucyte (*n* = 3). **E,** Cell-cycle distribution of PDM-272 organoid treated without or with Ceapin-A7 (3 μmol/L) after 2 days as determined by flow cytometric PI staining (*n* = 2). **F,** Gene expression of *ATF6*, Wnt signaling genes (*AXIN2*, *LGR5*, and *TCF7*), and Myc signaling genes (*MYC* and *CDCA7*) of PDM-272 organoid treated with Ceapin-A7 (3 μmol/L) for 2 days as determined by RT-qPCR (*n* = 3). **G,** Viability of PDM-272 after 7 days of growth in the presence of inactive Ceapin-A7 (3 μmol/L), or Ceapin-A7 (3 μmol/L; *n* = 3). **H,** Organoid confluence of PDM-272, as described in **G** (*n* = 3). **I,** Light microscopy images of PDM-272 organoid in **G** after 7-day treatment. **J,** PDM-272 were treated as in **G** for 2 days and were analyzed by RT-qPCR for Wnt signaling (*AXIN2*, *LGR5*, and *TCF7*) and Myc signaling (*MYC* and *CDCA7*) mRNA levels (*n* = 3). *, *P* < 0.05; **, *P* < 0.005; ***, *P* < 0.001; ****, *P* < 0.0001.

Despite its effectiveness as a selective inhibitor of ATF6 activation, Ceapin-A7 has poor pharmacokinetic properties, which limit its utility for *in vivo* implementation. We therefore turned to an *in vitro* model system that arguably has better pathophysiologic relevance to colorectal cancer than do xenografted cell lines, namely, cultured human intestinal organoids (90). We examined two normal and six malignant intestinal organoids. Although Ceapin-A7 did not impact the viability of the normal organoids, it decreased the viability and attenuated the growth of several malignant organoids (Fig. 6A and B). Further investigation using PDM-272 confirmed that Ceapin-A7 acted in a dose-dependent manner, restraining organoid growth within 2 days of addition (Fig. 6C and D). Moreover, a 2-day treatment with Ceapin-A7 attenuated cell-cycle progression (Fig. 6E) and downregulated mRNA expression of Wnt and Myc pathway target genes (Fig. 6F).

To verify inhibitor selectivity toward ATF6, we synthesized an inactive analog of Ceapin-A7 by modifying it with monomethyl ethylene glycol (inactive Ceapin-A7; Supplementary Fig. S6A). As predicted, the modified compound failed to block Tg-induced ATF6 processing in the PDM-272 organoid (Supplementary Fig. S6B). Importantly, only Ceapin-A7 and not its inactive analog attenuated PDM-272 viability and growth (Fig. 6G–I), in conjunction with the downregulation of *AXIN2*, *LGR5*, *TCF7*, *MYC*, and *CDCA7* mRNA (Fig. 6J). Taken together, these results demonstrate that attenuation of malignant intestinal organoid growth by selective pharmacologic inhibition of ATF6 occurs in concert with disruption of the Wnt and MYC signaling pathways.

To compare the relative importance of different UPR branches for organoid growth, we used specific small-molecule inhibitors of ATF6, IRE1, and PERK. As expected, in Tg-treated PDΜ-272 organoids, Ceapin-A7 attenuated ATF6 processing, whereas the IRE1 RNase inhibitor 4μ8C (91) curbed XBP1s generation and the PERK inhibitor AMG PERK 44 (92) inhibited CHOP and ATF4 induction (Supplementary Fig. S6C). However, whereas ATF6 inhibition attenuated PDM-272 viability and growth, IRE1 or PERK inhibition did not (Supplementary Fig. S6D–S6F). These results suggest a dependency of the PDM-272 organoid uniquely on ATF6 as compared with other UPR branches.

### ATF6 inhibition in organoids promotes broad intestinal differentiation

**Figure 7:**
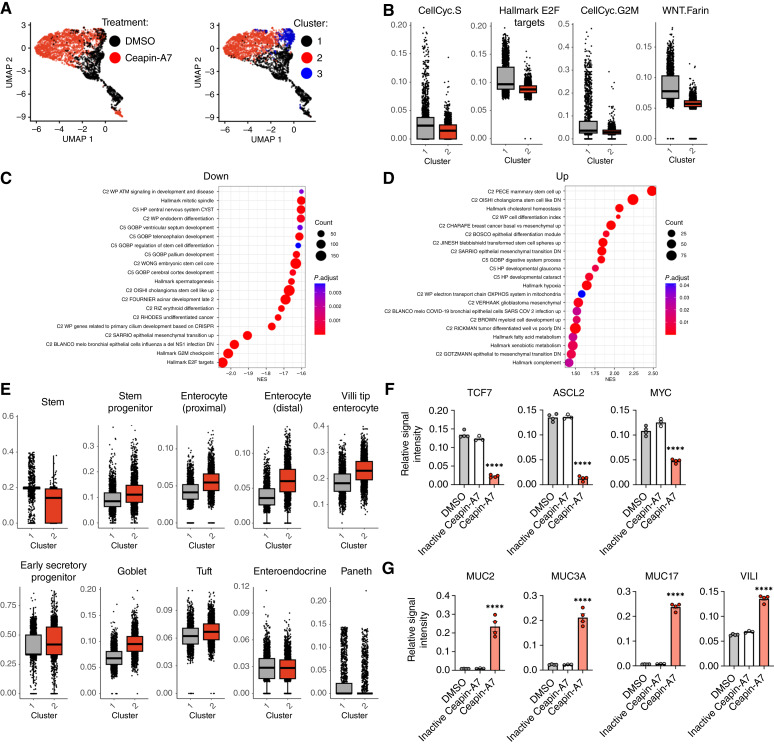
ATF6 inhibition in PDM-272 organoids attenuates cell-cycle progression and promotes multilineage intestinal differentiation. **A,** UMAPs based on principal components of all single-cell transcriptomes of PDM-272 organoid treated with DMSO (*n* = 2) or Ceapin-A7 (3 μmol/L; *n* = 2) for 2 days, as depicted by treatment condition (left) and cluster segmentation (right). **B,** Pathway enrichment scores of cluster 1 vs. cluster 2 described in **A**. **C,** Pathways most significantly downregulated in cluster 2 from cluster 1, as described in **A**. **D,** Pathways most significantly upregulated in cluster 2 from cluster 1, as described in **A**. **E,** UCell Signature Score of cluster 1 and cluster 2, as described in **A**. **F,** Effect of ATF6 inhibition on expression of proteins involved in Wnt signaling. PDM-272 organoid was incubated for 3 days with DMSO (*n* = 4), inactive Ceapin-A7 (3 μmol/L; *n* = 3), or ceapin-A7 (3 μmol/L; *n* = 4) and subjected to global proteome analysis, as described in detail in Materials and Methods. Relative signal intensity calculated based off of abundance of specified protein relative to total protein abundance per sample. **G,** Effect of ATF6 inhibition on expression of proteins involved in differentiation, as described in **F** (*n* ≥ 3). CellCyc, cell cycle; DN, downregulation; UP, upregulation. ****. *P* < 0.0001.

To gain further insights into the consequences of ATF6 inhibition in the organoid setting, we performed a scRNA-seq analysis of PDM-272, cultured in the absence or presence of Ceapin-A7. ATF6 disruption markedly changed the organoid’s cellular composition (Fig. 7A). Furthermore, ATF6 inhibition significantly downregulated genes associated with cell-cycle control and Wnt signaling (Fig. 7B and C), consistent with the cell line data. Among the most affected were the Cell-cycle and E2F target gene sets (Fig. 7C). In line with the other evidence for diminished stemness, ATF6 inhibition upregulated a number of gene sets related to intestinal cell differentiation (Fig. 7D), increasing differentiation scores for several intestinal cell types, including the tuft, goblet, and enterocyte lineages (Fig. 7E). A higher lineage score for one cell type did not preclude an increased score for another (Supplementary Fig. S7A), suggesting an uncoordinated differentiation of the organoid. In keeping with the absence of IRE1 and PERK cross-activation in cell lines, Ceapin-A7 did not significantly enrich the UPR/ER stress response gene sets (Supplementary Fig. S7B). It also did not upregulate the expression of apoptosis-related genes (Supplementary Fig. S7C), suggesting that ATF6 inhibition disrupts organoid growth by curbing proliferation and increasing differentiation rather than through programmed cell death.

To extend these transcriptomic findings, we performed a global proteomic analysis of PDM-272 organoids treated for 3 days with DMSO, inactive Ceapin-A7, or Ceapin-A7. As predicted from the scRNA-seq results, Ceapin-A7 but not its inactive analog downregulated protein sets involved in cell-cycle progression, most significantly including E2F, G2/M checkpoint, and Myc targets (Supplementary Fig. S7D). Wnt-pathway targets, including TCF7, ASCL2, and MYC, were also substantially depleted (Fig. 7F), whereas other *bona fide* targets like AXIN2 and LGR5 could not be detected. Conversely, proteins involved in goblet-cell function, namely MUC2, MUC3A, MUC17, and Villin (VILI), showed increased abundance (Fig. 7G), indicating onset of differentiation.

A small yet significant fraction of colorectal cancer cases displays genetic alterations that amplify cellular responsiveness to Wnt ligand-driven signaling rather than promote ligand-independent Wnt pathway activity. For example, damaging mutations of the ubiquitin E3 ligase RNF43 cause inappropriate stabilization of the RNF43 substrate Frizzled—a critical cell-surface coreceptor for Wnt (93). This stabilization enables sufficient signaling at lower Wnt ligand concentrations. PDM-272 harbors mutations in *RNF43* and its associated E3 ubiquitin ligase, *ZNRF3*, while lacking mutations in *APC* (Supplementary Table S4). The *RNF43* mutation leads to a stop-gain (R132*). This truncating mutation was found to be the only variant in a patient kindred with serrated polyposis, suggesting its pathogenicity (94). We reasoned that because PDM-272 can grow in the absence of exogenous Wnt supplementation, it may autonomously produce Wnt ligands(s) that are necessary for its growth and be hypersensitized to such ligands by its RNF43 inactivated status. Consistent with this notion, PDM-272 cell viability was strongly sensitive to small-molecule inhibition of the Wnt-specific acyltransferase Porcupine, required for Wnt maturation and secretion (LGK974; ref. 95), whereas an APC-mutant organoid, PDM-6, was much more resilient to Porcupine disruption (Supplementary Fig. S8A). ATF6 inhibition did not deplete components of the β-catenin destruction complex (APC, GSK3β), though it did enrich one component (AXIN1) while decreasing abundance of the total β-catenin protein (Supplementary Fig. S8B). Taken together with the loss of several β-catenin transcriptional targets, including TCF7, SLCL2, and MYC, these results indicate that ATF6 inhibition disrupts Wnt-pathway activity.

### Wnt surrogacy restores organoid growth under ATF6 inhibition

**Figure 8:**
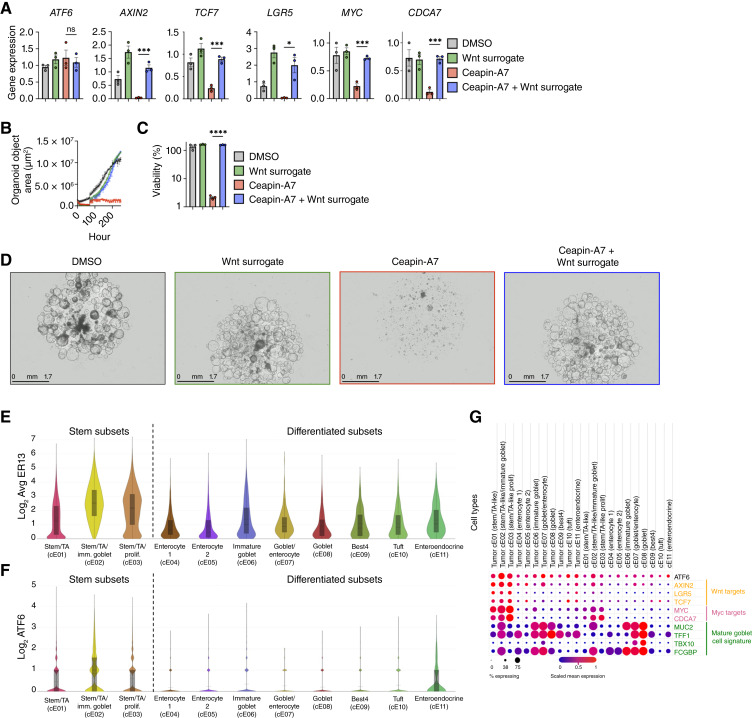
Wnt surrogate restores Wnt pathway activity and growth of PDM-272 organoids in context of ATF6 inhibition. **A,** mRNA gene expression as determined by RT-qPCR of *ATF6*, Wnt signaling genes (*AXIN2*, *LGR5*, and *TCF7*) and Myc signaling genes (*MYC* and *CDCA7*) of PDM-272 organoid treated with Wnt surrogate fusion protein (0.015 nmol/L) in the absence or presence of Ceapin-A7 (3 μmol/L) after 2 days (*n* = 3). **B,** Confluence of PDM-272 organoid treated with Wnt surrogate fusion protein (0.01 nmol/L) in the absence or presence of Ceapin-A7 (3 μmol/L) for 10 days (*n* = 3). **C,** Viability of PDM-272 organoid, as treated in **B** (*n* = 3). **D,** Light microscopy images of organoids in **B** after 10-day treatment. **E,** Expression of ER13 stratified οf colonic tumor epithelial cell subtypes and divided by stem subsets and differentiated subsets. **F,** Expression of *ATF6* as stratified cell subtypes described in **E**. **G,** Co-expression of *ATF6* and known Wnt signaling (*AXIN2*, *LGR5*, and *TCF7*) and Myc signaling (*MYC* and *CDCA7*) for various colon cancer epithelial cell subtypes. Avg, average; imm, immature; prolif, proliferation. *, *P* < 0.05; ***, *P* < 0.001.

Given that Wnt ligands display extensive diversity and complexity, we opted to test whether augmenting Wnt signaling with a recombinant Wnt mimetic could restore pathway activity and rescue organoid growth despite ATF6 inhibition. To this end, we used a previously engineered bispecific ligand, comprising Frizzled- and leucin-responsive regulatory protein (LRP)-binding domains fused to immunoglobulin fragment crystallizable (Fc), which acts as an effective and selective Wnt surrogate (96–98). A titration experiment showed that a sub-nmol/L concentration of the Wnt surrogate was sufficient to restore PDM-272 growth during ATF6 inhibition (Supplementary Fig. S8C). Consistently, although having no effect on ATF6 expression, Wnt-surrogate treatment of PDM-272 organoids in the context of Ceapin-A7 treatment markedly restored mRNA expression of *AXIN2*, *TCF7*, *LGR5*, *MYC*, and *CDCA7* (Fig. 8A). Strikingly, Wnt surrogacy substantially protected organoid viability and growth under ATF6 inhibition over a 10-day treatment (Fig. 8B–D). In contrast, Wnt surrogacy did not restore viability of the *APC*-mutant organoids PDM-6 or PDM-96 upon ATF6 inhibition with Ceapin-A7 (Supplementary Fig. S8D) nor upon ATF6 depletion in Colo201 and CCK81 cells (Supplementary Fig. S8E), which possess *APC* mutations that likely circumvent Wnt ligand dependency.

To explore the clinical relevance of our finding that ATF6 contributes to colorectal cancer stemness, we analyzed ER13 expression across 11 defined colorectal cancer epithelial cell subsets (40) -consisting of stem compartments, including transit amplifying cells, and differentiated lineages, i.e., goblet and enterocyte. Tumor cells within the stem subtypes had higher ER13 expression than did those within the differentiated compartments (Fig. 8E; Supplementary Fig. S8F), and *ATF6* displayed a similar expression pattern to ER13 (Fig. 8F; Supplementary Fig. S8G). Moreover, these same *ATF6*-enriched stem compartments had elevated expression of Wnt- and Myc-signaling target genes, whereas differentiated cell compartments had lower levels of these target genes (Fig. 8G). Taken together, these results support the conclusion that ATF6 mediates colorectal cancer stemness by facilitating oncogenic Wnt and Myc signaling.

## Discussion

This study is the first to demonstrate that interference against ATF6 activity significantly reduces growth of diverse colorectal cancer models *in vitro* and *in vivo*. We provide evidence that the mechanism underlying tumor-growth defects conferred by ATF6 disruption involves attenuation of Wnt and Myc signaling, reduced cell-cycle progression and stemness, and increased differentiation. We identify ATF6 as an important facilitator of oncogenic signaling in colorectal cancer, and as such, an exciting potential therapeutic target.

We uncovered elevated ATF6 activity based on the ER13 gene signature in several prevalent solid cancers, including colorectal cancer, on which we focused in this present study. We examined the effect of interference with ATF6 activity on growth of human colorectal cancer cell lines and organoids. We used diverse strategies to disrupt ATF6, namely, genetic silencing via shRNA, CRISPR, or ASO, and small-molecule inhibition with Ceapin-A7. All described ATF6 interventions were effective in various models at reducing cell viability and proliferation, providing the first evidence that ATF6 directly facilitates colorectal cancer growth. ATF6 dependency occurred in the absence of exogenous ER stressors, implicating ATF6 as a constitutive intrinsic facilitator of colorectal cancer growth. Despite the known interconnectedness between UPR branches (99), ATF6 interference did not promote compensatory activation of IRE1 or PERK, suggesting a more specialized role for ATF6 in colorectal cancer than seen in the canonical context of drug-induced ER stress. ATF6 disruption led to an increase in CDKi expression and to cell-cycle inhibition independent of apoptosis activation. Bulk RNA-seq analysis confirmed this by showing downregulation of cell cycle S-phase and E2F gene sets and further revealed that ATF6 silencing reduces Myc and Wnt signaling. Consistent with the latter were a decrease in stemness and an increase in differentiation gene-et expression. Strikingly, the disruption of Wnt signaling and its downstream consequences occurred within the first day of induced ATF6 silencing. This immediacy suggests that ATF6 loss augments Wnt signaling directly rather than indirectly, as a secondary consequence of other events. A core feature of stemness, namely, seeding potential, was altered similarly upon silencing of either ATF6 or β-catenin, supporting the conclusion that ATF6 regulates stemness by augmenting Wnt signaling. Importantly, malignant intestinal organoids also showed varying sensitivity to ATF6 inhibition, demonstrating a potential clinical relevance of our findings. We investigated the PDM-272 organoid in greater depth. Sensitivity of this organoid was specific to ATF6 inhibition as compared with the other UPR branches, IRE1 and PERK. Reduced PDM-272 growth coincided with disrupted Wnt signaling and with unbiased multilineage differentiation. Restoring pathway activation by a Wnt surrogate was sufficient to reverse the impact of ATF6 inhibition on PDM-272, validating the conclusion that ATF6 regulates Wnt signaling in this model.

Perhaps complementing our observations, previous work using cytomegalovirus early enhancer/chicken β-actin (CAG) promoter–driven ectopic overexpression of nATF6 uncovered an indirect role for ATF6 in colorectal cancer tumorigenesis (100). In that context nATF6 overexpression in the gut promoted colonic tumorigenesis by eroding the mucin barrier and thereby enabling bacterial invasion of intestinal crypts (100). In a separate work, and in contrast to our present findings, overexpression of nATF6 inhibited colorectal cancer cell line growth and stemness (101). However, yet another study demonstrated that CAG promoter–based overexpression of nATF6 drives nonphysiologic levels of ATF6 activity (36). Indeed, loss-of-function strategies are less prone to artifact and arguably provide a more reliable approach to investigate clinically relevant roles of ATF6. In this vein, CRISPR interreference of ATF6 in HCT116 cells required concomitant deletion of IRE1α to reduce tumor growth (102), which indicates that both UPR branches can play a role at least in some circumstances. Notwithstanding, in Colo201 cells, CRISPR-based ATF6 deletion did not reduce viability as effectively as inducible silencing or chemical inhibition, suggesting that CRISPR interference with ATF6 may drive compensatory adaptation.

The finding that ATF6 silencing inhibits cell-cycle progression and increases CDKi expression in colorectal cancer cells is consistent with recent evidence that ATF6 deletion in pancreatic β-cells during insulitis leads to cell-cycle arrest and increases the expression of *CDKN1A* (103). This upregulation of *CDKN1A* may be due to an association of ATF6 with CREB regulated transcription coactivator 2 that augments its binding to the *CDKN1A* promoter (104).

Our studies reveal that ATF6 supports oncogenic Wnt and Myc signaling. Silencing of *ATF6* phenocopied loss of β-catenin while decreasing stem gene-set expression, cell viability, and seeding capacity. Previous work suggests that ER-stress induction through perturbation of BiP/GRP78 (*HSPA5*) in colorectal cancer cells and colonic organoids opposes stemness and reduces the expression of Wnt target genes such as *LGR5* (17, 18). These results may be akin to nATF6 or XBP1s overexpression studies, which also report curbed stem gene expression, as both approaches evoke a hyperactivated ER stress response. Importantly, selective interference with ATF6 did not increase general ER stress, and inhibition of IRE1 or PERK had little impact on PDM-272 organoid growth, suggesting that the loss of colorectal cancer stemness upon ATF6 disruption is specific and independent of global ER stress. A recent report finds that XBP1s acts on the promoter region of *LEF1* to support Wnt signaling in hepatocellular carcinoma (105). We observed decreased *LEF1* expression in Colo201 and CCK81 ATF6-silenced cells despite a lack of significant changes in XBP1s. Similarly, PDM-272 organoids showed no sensitivity to IRE1α RNase inhibition, which would prevent XBP1s production. These results suggest that ATF6 supports Wnt signaling in colorectal cancer independently of XBP1s, inviting future consideration that nATF6 may directly act on Wnt pathway target genes or potentially as a cofactor to β-catenin. Notably, ATF6 knockdown did not impact *CTNNB1* mRNA levels (Supplementary Fig. S8H).

It has been reported that nonmalignant organoid differentiation requires PERK signaling, whereas ATF6 or IRE1 silencing in a model cell line does not restore stem gene expression lost upon ER stress (17). A separate study found that treatment of embryonic stem cells with a small-molecule compound that facilitates ATF6 trafficking induced mesodermal differentiation not through Wnt signaling but rather through ER expansion, opposed by concomitant Ceapin-A7 treatment (106). Together, these studies suggest that ATF6 interference should not impact nonmalignant embryonic or colonic stemness, which is also supported by the observation that ATF6α deletion is well tolerated during mouse postnatal development at least up to 7 months of age (107). Consistent with this notion, Ceapin-A7 treatment did not disrupt the growth of two normal tissue organoids. By contrast, it promoted unbiased terminal differentiation and loss of viability of the PDM-272 colorectal cancer organoid, suggesting that the tumorigenic stem compartment may co-opt ATF6 to support Wnt signaling and cell pluripotency. Supporting this latter idea, we discerned enriched expression of *ATF6* and ER13 in the tumor stem/transit-amplifying cell compartment and diminished expression in differentiated epithelial cell lineages of colorectal cancer samples. Importantly, there was little ER13 activity in healthy colonic samples, suggesting that ATF6 may become co-opted upon acquisition of dysregulating mutations in the Wnt pathway and that targeting ATF6 may be well tolerated by nonmutated cells in the context of disease. Nevertheless, preclinical validation in relevant safety models will doubtlessly be required.

Loss of Wnt signaling and stem gene expression occurred in *APC*-mutant (Colo201, CCK81) and *APC-*wild-type (PDM-272) settings. In the latter instance, we inferred that a pathogenic stop-gain mutation in *RNF43* (R132*), which occurs in nearly 10% of colorectal cancer cases with *RNF43* mutations (108), facilitates Wnt ligand-dependent growth. This was confirmed by the strong sensitivity of PDM-272 to Porcupine inhibition. In this context, restoring signaling with the Wnt-surrogate preserved organoid viability despite ATF6 inhibition. Mutations that augment Wnt ligand activity, e.g., in the LGR5 ligand *RSPO*, are mutually exclusive to *APC* mutations, illustrating two alternative pathways for oncogenic Wnt signaling in colorectal cancer (109). The inhibitory effect of ATF6 disruption on growth of several *APC*-mutant cell lines and organoids suggests that ATF6 may also augment ligand-independent Wnt pathway activity. However, the specific mechanisms underlying ATF6’s augmentation of the Wnt pathway in either ligand-dependent or -independent settings have yet to be elucidated. Optimal modulation of Wnt pathway activity seems critical to colorectal cancer survival, as overexpression of *CTNNB1* in *APC*-mutant cell lines is synthetically lethal (110). A cooperative relationship between Wnt and Myc is well described (75). For example, a SNP insertion within the *MYC* promoter suppresses intestinal tumorigenesis in APC-mutant mice (111). Notably, ATF6 silencing disrupted both Wnt and Myc signaling, underscoring the dual potential of ATF6 for therapeutic targeting in colorectal cancer.

Earlier reports have ascribed roles for ATF6 in malignancy beyond colorectal cancer. Loss of *p53* in triple-negative breast cancer may activate ATF6 processing (69), and dormant squamous carcinoma cells leverage ATF6 to maintain survival *in vitro* and *in vivo* (112). We found increased ER13 activity in several solid cancer tissues, which suggests that ATF6 may be co-opted in other malignancies as well. Two notable cancers with elevated ER13 expression that also display varying dependency on Wnt signaling are lung (113) and breast (114). Regardless, it is conceivable that the tumor promoting role of ATF6 extends beyond Wnt signaling, for example, in protecting against DNA damage (70). Our report marks an important advance in establishing ATF6 as a compelling novel target for the treatment of cancer.

## Supplementary Material

Supplementary Figure S1 CRC tumors display elevated expression of ATF6 target genes

Supplementary Figure S2 Disruption of ATF6 attenuates growth of multiple CRC cell lines in vitro and in vivo

Supplementary Figure S3 ATF6 disruption attenuates cell cycle progression and upregulates specific CDK inhibitors

Supplementary Figure S4 ATF6 disruption decreases CRC cell seeding capacity

Supplementary Figure S5 β-catenin knockdown phenocopies ATF6 silencing

Supplementary Figure S6 Selective inhibition of ATF6 but not IRE1 or PERK attenuates PDM-272 organoid growth

Supplementary Figure S7 ATF6 inhibition in PDM-272 organoids attenuates cell cycle progression and promotes multilineage intestinal differentiation

Supplementary Figure S8 Wnt surrogate restores Wnt-pathway activity and growth of PDM-272 organoids in context of ATF6 inhibition

Supplementary Table S1 Comparison of ER13 and ER16 gene signatures

Supplementary Table S2 BLAST analysis of ATF6 shRNAs

Supplementary Table S3 Materials Table

Supplementary Table S4 Annotation of CRC organoid mutations
