# Supplementary material for: ATF6 Promotes Colorectal Cancer Growth and Stemness by Regulating the Wnt Pathway
Source: Cancer Res Commun. 2024 Oct 21;4(10):2734–55. doi: 10.1158/2767-9764.CRC-24-0268 (PMC11492184; doi:10.1158/2767-9764.CRC-24-0268)
Supplement: Supplementary Figure S1 — CRC tumors display elevated expression of ATF6 target genes [file crc-24-0268_supplementary_figure_s1_supps1.pdf]

Figure S1

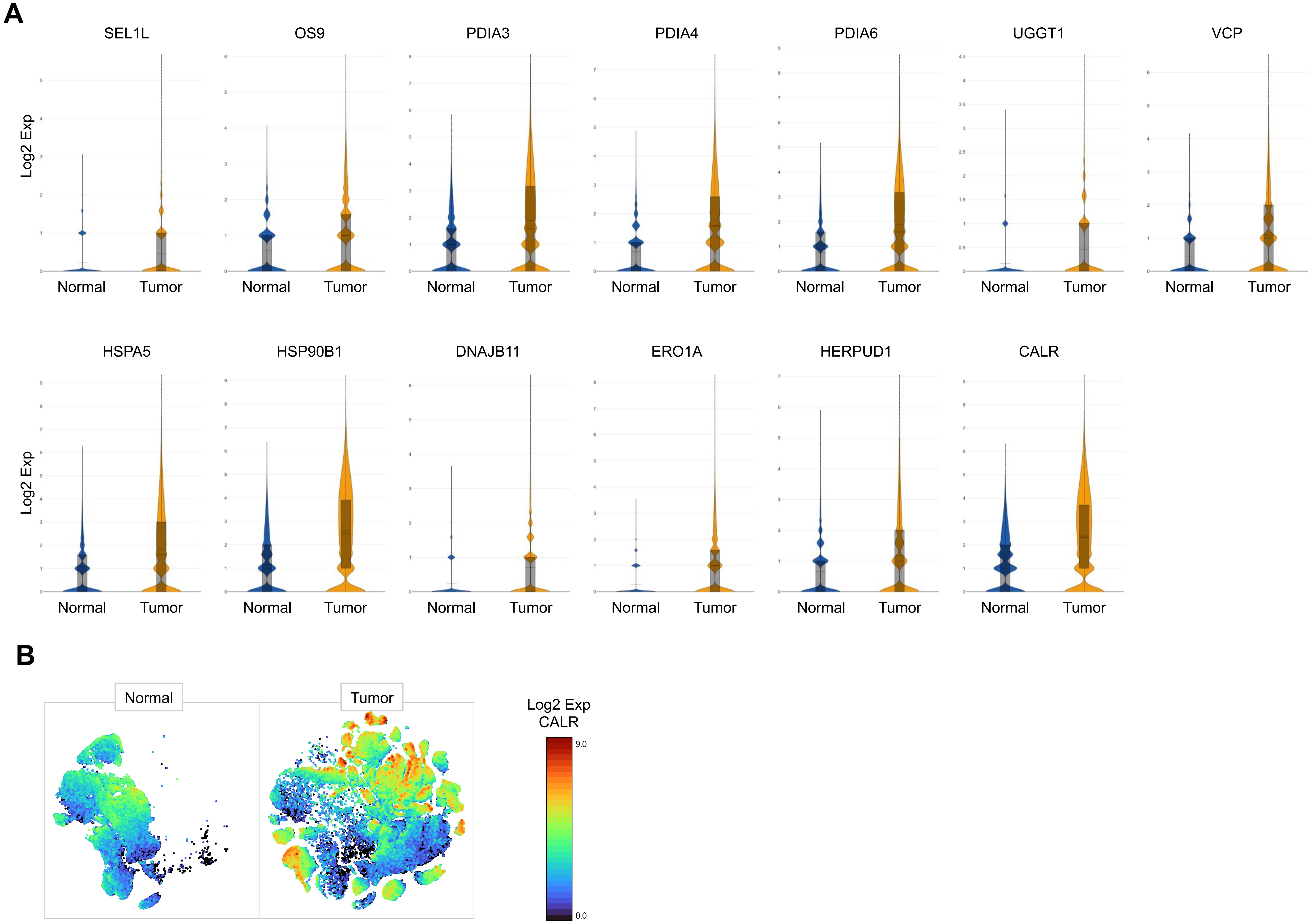
A
SEL1L
OS9
PDIA3
PDIA4
PDIA6
UGGT1
VCP
HSPA5
HSP90B1
DNAJB11
ERO1A
HERPUD1
CALR
Log2 Exp
Normal
Tumor
B
Normal
Tumor
Log2 Exp
CALR
9.0
0.0

Figure S1: CRC tumors display elevated expression of ATF6 target genes

(A) Expression (Exp) of specified ER13 genes in Normal and Tumor colonic cells.

(B) Cellular distribution and expression of ER13 gene *CALR* in Normal and Tumor colonic cells.
