# Supplementary material for: ATF6 Promotes Colorectal Cancer Growth and Stemness by Regulating the Wnt Pathway
Source: Cancer Res Commun. 2024 Oct 21;4(10):2734–55. doi: 10.1158/2767-9764.CRC-24-0268 (PMC11492184; doi:10.1158/2767-9764.CRC-24-0268)
Supplement: Supplementary Figure S2 — Disruption of ATF6 attenuates growth of multiple CRC cell lines in vitro and in vivo [file crc-24-0268_supplementary_figure_s2_supps2.pdf]

Figure S2

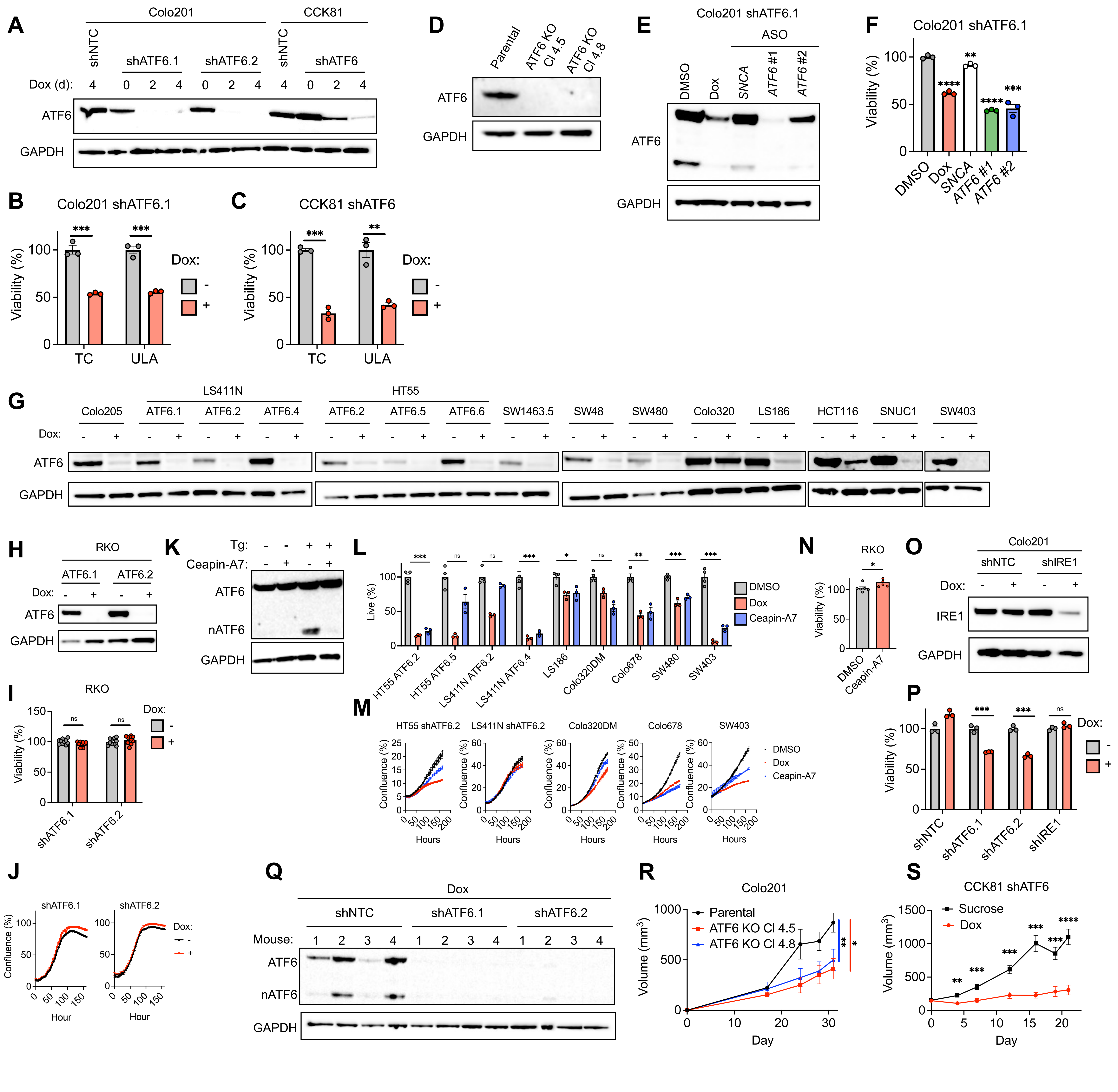

A
Colo201
CCK81
shNTC
shATF6.1
shATF6.2
shATF6
Dox (d):
ATF6
GAPDH
B
Colo201 shATF6.1
Viability (%)
TC
ULA
Dox:
C
CCK81 shATF6
D
Parental
ATF6 KO Cl 4.5
ATF6 KO Cl 4.8
E
Colo201 shATF6.1
ASO
DMSO
Dox
SNCA
ATF6 #1
ATF6 #2
F
G
Colo205
LS411N
HT55
ATF6.1
ATF6.2
ATF6.4
ATF6.5
ATF6.6
SW1463.5
SW48
SW480
Colo320
LS186
HCT116
SNUC1
SW403
H
RKO
I
K
Tg:
Ceapin-A7:
nATF6
L
Live (%)
Ceapin-A7
HT55 ATF6.2
HT55 ATF6.5
LS411N ATF6.2
LS411N ATF6.4
LS186
Colo320DM
Colo678
SW480
SW403
M
HT55 shATF6.2
LS411N shATF6.2
Colo320DM
Colo678
SW403
Confluence (%)
Hours
N
O
Colo201
shNTC
shIRE1
IRE1
P
J
shATF6.1
shATF6.2
Hour
Q
Mouse:
R
Colo201
Parental
ATF6 KO Cl 4.5
ATF6 KO Cl 4.8
Volume (mm³)
Day
S
CCK81 shATF6
Sucrose
Dox

Figure S2: Disruption of ATF6 attenuates growth of multiple CRC cell lines *in vitro* and *in vivo*

- **(A)** Validation of ATF6 knockdown in Colo201 and CCK81 cells by immunoblot (IB) analysis after specified day (d) of Doxycycline (Dox) treatment. For shATF6, two independent clones for Colo201 were established and one pooled line for CCK81. Pooled lines for shNTC were established for both Colo201 and CCK81.
- **(B)** Viability of Colo201 shATF6.1 cell lines grown in the absence or presence of Dox on standard tissue culture (TC) (left) or ultralow attachment ULA (right) plates (n=3) after 7 days.
- **(C)** Viability of CCK81 shATF6 cell lines treated as in **B** (n=3).
- **(D)** Validation of CRISPR/Cas9-based ATF6 knockout (KO) in Colo201 cells by IB. Parental Colo201 (WT) and two independent KO clones (Cl 4.5, Cl 4.8) are shown.
- **(E)** Validation of ATF6 knockdown by antisense oligonucleotides (ASOs). Colo201 shATF6.1 cell were treated with Dox (0.5 μg/ml) or with lipid nanoparticles (LNPs) containing one of two separate ASOs against ATF6 (*ATF6* #1, *ATF6* #2) (30 μg/ml) or with LNPs containing ASOs against negative control α-synuclein (*SNCA*) (30 μg/ml) and analyzed by IB after 2 days.
- **(F)** Viability comparison of Dox-induced or ASO-mediated ATF6 interference. Colo201 shATF6.1 cells were treated with Dox or LNPs containing ASOs against *SNCA* or *ATF6* as in **E** and analyzed for viability after 5 days (n=3).
- **(G)** Validation of ATF6 knockdown in additional CRC cell lines. Lines were treated for 3 days in absence (-) or presence (+) of Dox (0.5 μg/ml). Three clones each are shown for LS411N and HT55, while pools are shown for the other cell lines.
- **(H)** Validation of Dox-induced ATF6 knockdown in two RKO cell clones after 2 days of Dox treatment.
- **(I)** Viability RKO shATF6 cell lines after 7 days of Dox treatment (n=10).
- **(J)** Proliferation of RKO shATF6 cell lines grown in absence or presence of Dox by Incucyte as in **I**.
- **(K)** Inhibition of ATF6 activation by Ceapin-A7. Colo201 cells were treated with Thapsigargin (Tg, 100 nM) in the absence or presence of Ceapin-A7 (10 μM) for 3 h and analyzed for abundance of the processed form of ATF6, nuclear ATF6 (nATF6), by IB.

(L) Comparison of Dox-induced ATF6 knockdown and Ceapin-A7-based inhibition. Indicated cell lines expressing Dox-inducible ATF6 shRNA were analyzed for viability after 7 days of treatment with Dox (0.5 µg/ml) or Ceapin-A7 (10 µM) (n≥3).

(M) Cells were treated as in **L** and analyzed for proliferation by Incucyte.

(N) Viability of RKO cells treated with Ceapin-A7 (10 µM) for 7 days (n≥5).

(O) Validation of Dox-induced IRE1 knockdown in Colo201 cells by IB analysis after a 2-day Dox treatment (0.5 µg/ml).

(P) Comparison of viability upon Dox-induced knockdown of ATF6 or IRE1 in Colo201 cells. Cells expressing inducible shNTC, shATF6, or shIRE1 were treated with Dox (0.5 µg/ml) and analyzed for viability after 7 days.

(Q) Validation of ATF6 knockdown in vivo. Mice bearing Colo201 tumors expressing inducible shNTC or shATF6 were offered water supplemented with 5% sucrose and Dox (0.5 mg/ml) for 10 days. Tumors were collected from individual mice (n=4) and analyzed by IB.

(R) Tumor growth kinetics of parental Colo201 (WT) or ATF6 KO clones (Cl) 4.5 and 4.8 (n=10 per group). Tumor growth at Day 31 were performed using Student's t-test between WT and 4.5, 4.8 tumors.

(S) Tumor growth kinetics of CCK81 shATF6 tumors. Once subcutaneous inoculated cells established palpable tumors, mice were offered water supplemented with 5% sucrose or 5% sucrose containing Dox (0.5 mg/ml) on monitored for 21 days, as described in **Fig. 2L** (n≥10 per group).
