# Supplementary material for: ATF6 Promotes Colorectal Cancer Growth and Stemness by Regulating the Wnt Pathway
Source: Cancer Res Commun. 2024 Oct 21;4(10):2734–55. doi: 10.1158/2767-9764.CRC-24-0268 (PMC11492184; doi:10.1158/2767-9764.CRC-24-0268)
Supplement: Supplementary Figure S3 — ATF6 disruption attenuates cell cycle progression and upregulates specific CDK inhibitors [file crc-24-0268_supplementary_figure_s3_supps3.pdf]

Figure S3

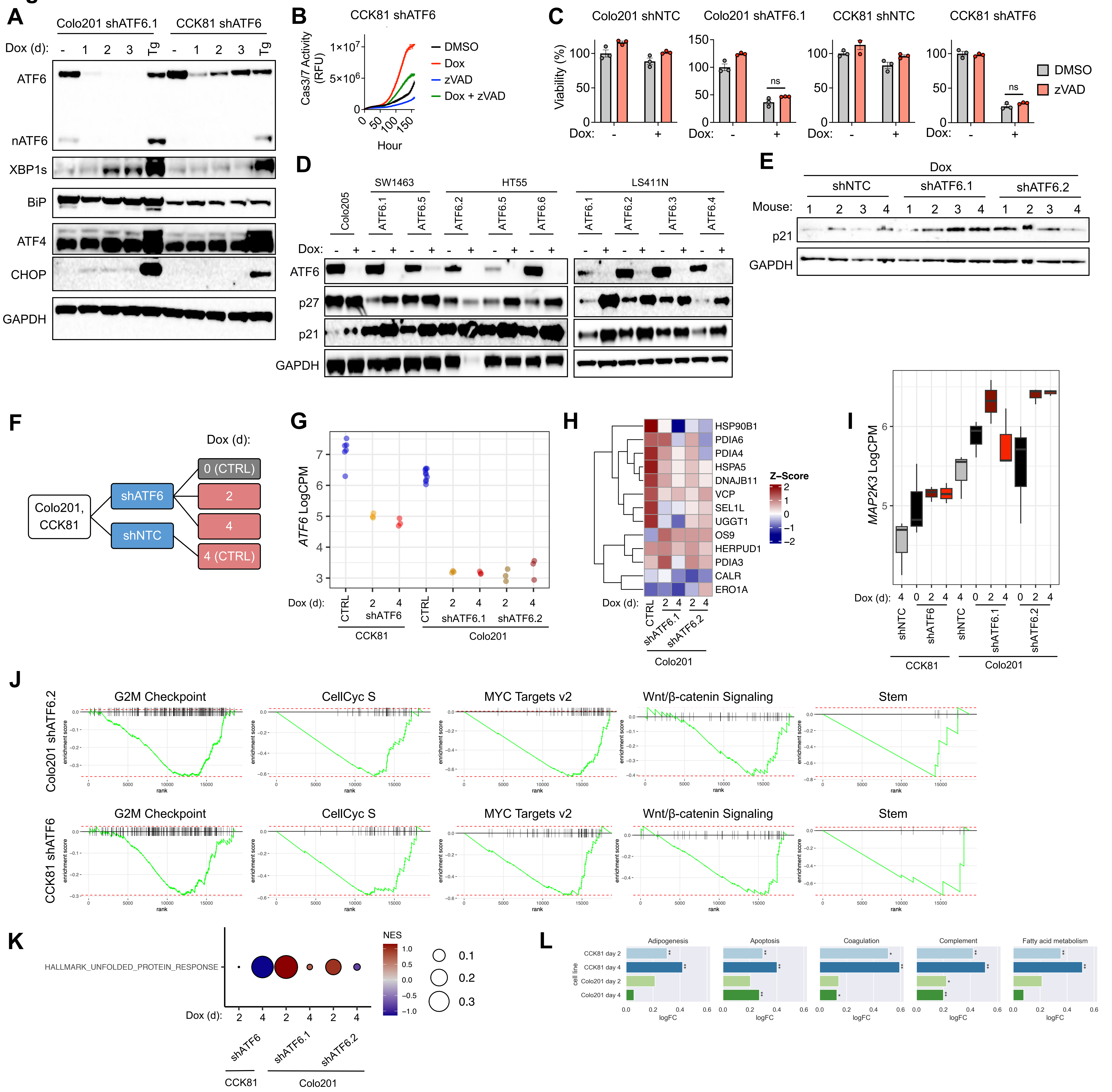

A
Colo201 shATF6.1
CCK81 shATF6
Dox (d): - 1 2 3 Tg - 1 2 3 Tg
ATF6
nATF6
XBP1s
BiP
ATF4
CHOP
GAPDH
B
CCK81 shATF6
Cas3/7 Activity (RFU)
Hour
DMSO
Dox
zVAD
Dox + zVAD
C
Colo201 shNTC
Colo201 shATF6.1
CCK81 shNTC
CCK81 shATF6
Viability (%)
Dox:
ns
DMSO
zVAD
D
Colo205
SW1463
HT55
LS411N
ATF6.1
ATF6.5
ATF6.2
ATF6.6
ATF6.3
ATF6.4
Dox:
ATF6
p27
p21
GAPDH
E
Dox
shNTC
shATF6.1
shATF6.2
Mouse:
p21
GAPDH
F
Dox (d):
Colo201, CCK81
shATF6
shNTC
0 (CTRL)
2
4
4 (CTRL)
G
ATF6 LogCPM
Dox (d):
CTRL
shATF6
shATF6.1
shATF6.2
CCK81
Colo201
H
HSP90B1
PDIA6
PDIA4
HSPA5
DNAJB11
VCP
SEL1L
UGGT1
OS9
HERPUD1
PDIA3
CALR
ERO1A
Z–Score
Dox (d):
CTRL
shATF6.1
shATF6.2
Colo201
I
MAP2K3 LogCPM
Dox (d):
shNTC
shATF6
shATF6.1
shATF6.2
CCK81
Colo201
J
Colo201 shATF6.2
CCK81 shATF6
G2M Checkpoint
CellCyc S
MYC Targets v2
Wnt/β-catenin Signaling
Stem
enrichment score
rank
K
HALLMARK_UNFOLDED_PROTEIN_RESPONSE
NES
Dox (d):
shATF6
shATF6.1
shATF6.2
CCK81
Colo201
L
Adipogenesis
Apoptosis
Coagulation
Complement
Fatty acid metabolism
cell line
CCK81 day 2
CCK81 day 4
Colo201 day 2
Colo201 day 4
logFC

Figure S3: ATF6 disruption attenuates cell cycle progression and upregulates specific CDK inhibitors

- **(A)** IB analysis of UPR proteins in Colo201 shATF6.1 and CCK81 shATF6 cell lines treated with Dox for specified length of time or Tg (100 nM) for 4 h. Cells not treated Dox (-) were harvested after 3 days.
- **(B)** Caspase-3/7 activity of CCK81 shATF6 cells treated with Dox in the absence or presence of the pan-caspase inhibitor zVAD (20 μM) as quantified by Incucyte detection of Caspase-3/7 substrate conversion-based fluorescence (Raw Fluorescent Units (RFUs)) (n=3).
- **(C)** Viability of Colo201 shNTC, shATF6 and CCK81 shNTC, shATF6 cell lines treated with Dox in the absence or presence of zVAD (20 μM) for 7 days (n=3).
- **(D)** IB analysis of p21 and p27 protein levels in various shATF6 cell lines after 3 day treatment with Dox treatment (0.5 μg/ml).
- **(E)** IB analysis of p21 protein levels in established Colo201 shNTC, shATF6.1, or shATF6.2 tumors from mice placed on water containing sucrose (5%) and Dox (0.5 mg/ml) for 10 days.
- **(F)** Schematic design of RNA-Seq study of Colo201 shNTC, shATF6.1, shATF6.2 and CCK81 shNTC, shATF6 lines treated with Dox for 2 and 4 days.
- **(G)** Verification of *ATF6* mRNA depletion in Colo201 and CCK81 shATF6 cell lines while unchanged in control (CTRL) conditions (shNTC Day 4, shATF6 Day 0).
- **(H)** Heatmap for ER13 genes as indicated in Colo201.
- **(I)** Verification of no decreased expression of *MAP2K3* mRNA in Colo201 and CCK81 shATF6 cell lines upon Dox treatment.
- **(J)** Representative GSEA trace plots of various indicated gene sets of Colo201 shATF6.2 cells (top row) and CCK81 cells (bottom row).
- **(K)** GSEA plot of Hallmark Unfolded Protein Response (UPR) gene set.
- **(L)** GSEA increases (log2Fold Change(FC)) for Adipogenesis, Apoptosis, Coagulation, Complement, and Fatty acid metabolism Hallmark gene sets.
