# Supplementary material for: ATF6 Promotes Colorectal Cancer Growth and Stemness by Regulating the Wnt Pathway
Source: Cancer Res Commun. 2024 Oct 21;4(10):2734–55. doi: 10.1158/2767-9764.CRC-24-0268 (PMC11492184; doi:10.1158/2767-9764.CRC-24-0268)
Supplement: Supplementary Figure S4 — ATF6 disruption decreases CRC cell seeding capacity [file crc-24-0268_supplementary_figure_s4_supps4.pdf]

Figure S4

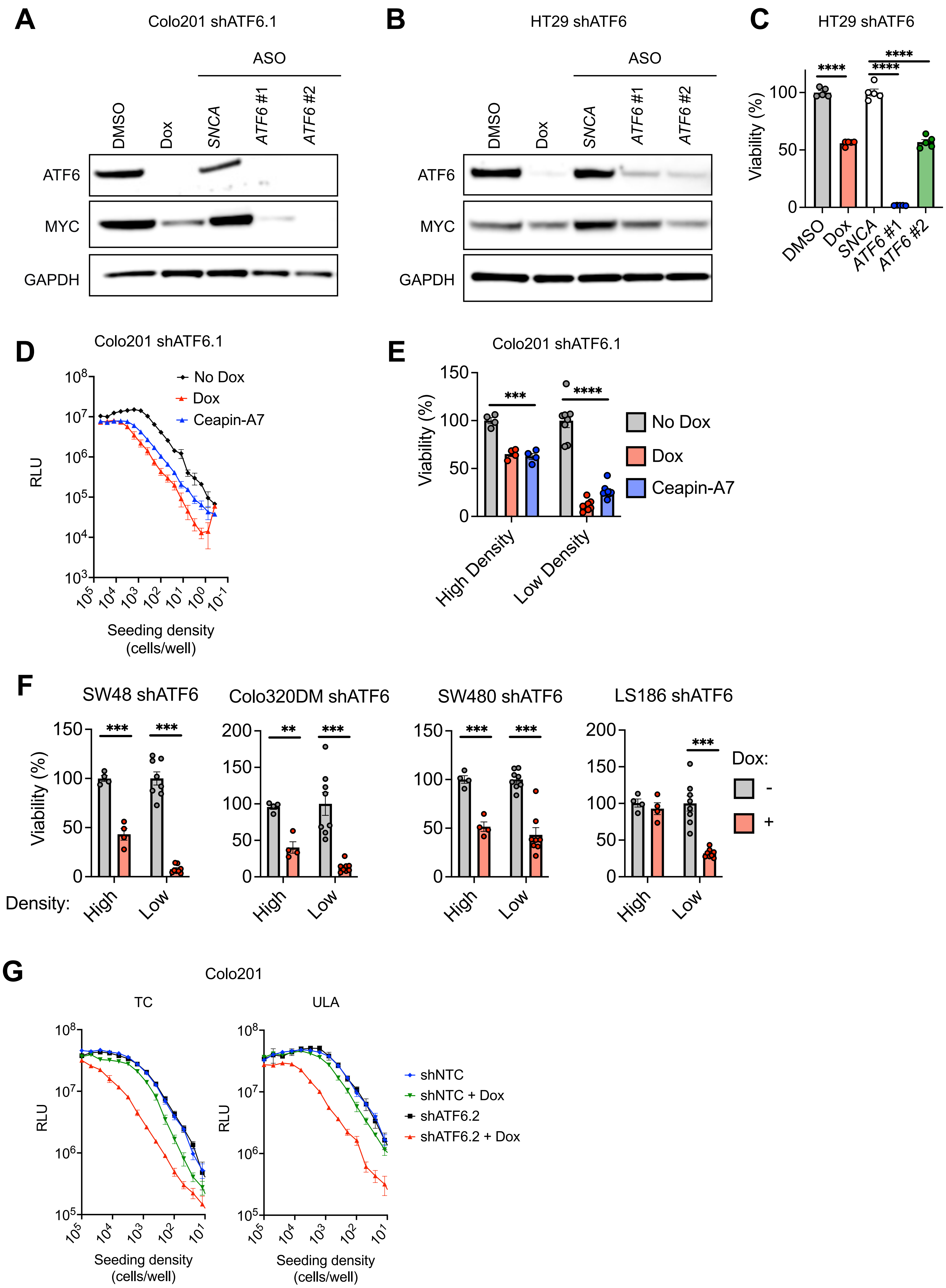
A
Colo201 shATF6.1
ASO
DMSO
Dox
SNCA
ATF6 #1
ATF6 #2
ATF6
MYC
GAPDH
B
HT29 shATF6
ASO
DMSO
Dox
SNCA
ATF6 #1
ATF6 #2
ATF6
MYC
GAPDH
C
HT29 shATF6
Viability (%)
****
****
****
DMSO
Dox
SNCA
ATF6 #1
ATF6 #2
D
Colo201 shATF6.1
No Dox
Dox
Ceapin-A7
RLU
Seeding density
(cells/well)
E
Colo201 shATF6.1
Viability (%)
***
****
High Density
Low Density
No Dox
Dox
Ceapin-A7
F
SW48 shATF6
Colo320DM shATF6
SW480 shATF6
LS186 shATF6
Viability (%)
Density:
High
Low
Dox:
-
+
G
Colo201
TC
ULA
RLU
Seeding density
(cells/well)
shNTC
shNTC + Dox
shATF6.2
shATF6.2 + Dox

Figure S4: ATF6 disruption decreases CRC cell seeding capacity

- **(A)** IB of Colo201 shATF6.1 cells treated for 3 days with Dox (0.5 µg/ml) or LNP containing ASOs (30 µg/ml) for specified gene.
- **(B)** IB of HT29 shATF6 cells treated as described in **A**.
- **(C)** Viability of HT29 shATF6 cells treated for 6 days with conditions described **A**. Viability normalized to DMSO control for Dox condition and normalized to *SNCA* for *ATF6* #1, *ATF6* #2 ASO treatments (n=5).
- **(D)** Cell abundance as determined by relative luminescence units (RLU) assay of Colo201 shATF6.1 cells initially seeded at specified density and grown with or without Dox (0.5 µg/ml) or Ceapin-A7 (10 µM) for 7 days (n≥3).
- **(E)** Relative viability of Colo201 shATF6.1 plated at High Density (1.25e4 cells/well) or low density (2e2 cells/well) as treated in **D** (n≥3)**.**
- **(F)** Viability of SW48 shATF6, Colo320DM shATF6, SW480 shATF6, LS186 shATF6 cells that were initially seeded at high density (1.25e4 cell/well) or low density (8e2 cell/well) and grown in presence or absence of Dox (0.5 µg/ml) for 7 days (n≥4)**.**
- **(G)** Cell abundance as determined by RLU of Colo201 shNTC and shATF6.2 cells initially seeded at specified density on tissue culture (TC) or ultralow attachment (ULA) 3D well plates and grown with or without Dox (0.5 µg/ml) for 7 days.
