# Supplementary material for: ATF6 Promotes Colorectal Cancer Growth and Stemness by Regulating the Wnt Pathway
Source: Cancer Res Commun. 2024 Oct 21;4(10):2734–55. doi: 10.1158/2767-9764.CRC-24-0268 (PMC11492184; doi:10.1158/2767-9764.CRC-24-0268)
Supplement: Supplementary Figure S5 — β-catenin knockdown phenocopies ATF6 silencing [file crc-24-0268_supplementary_figure_s5_supps5.pdf]

Figure S5

A

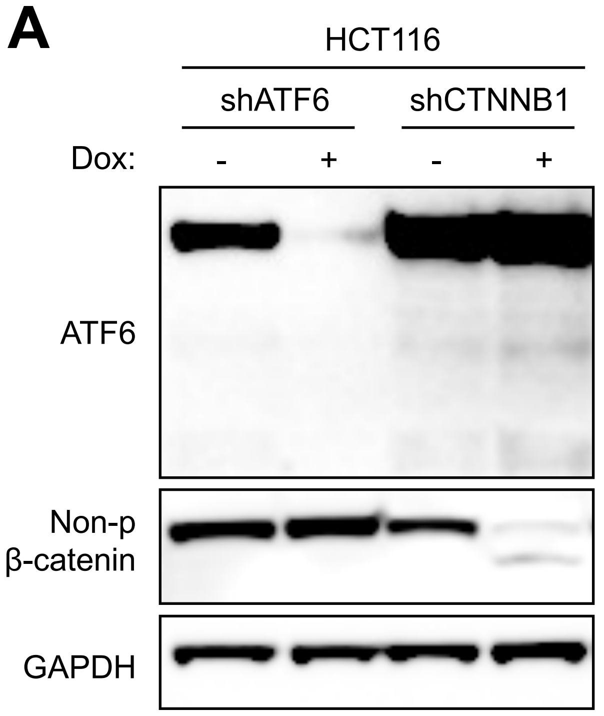

B

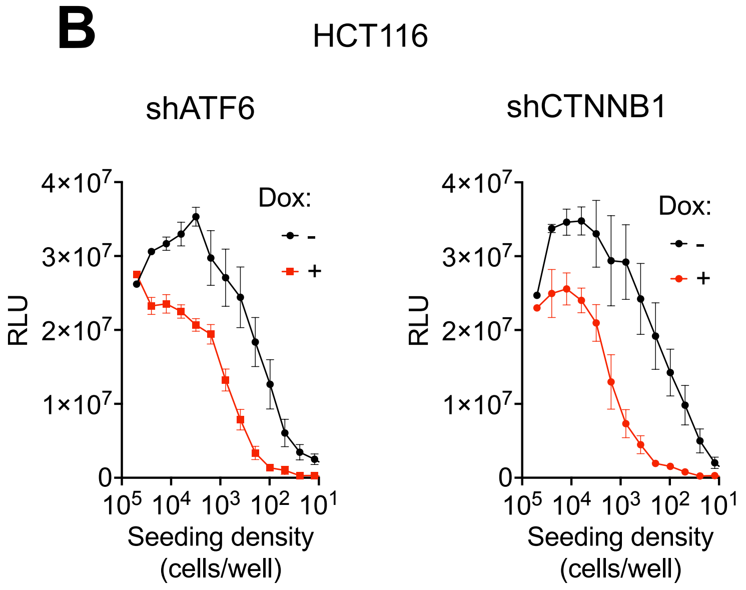

C

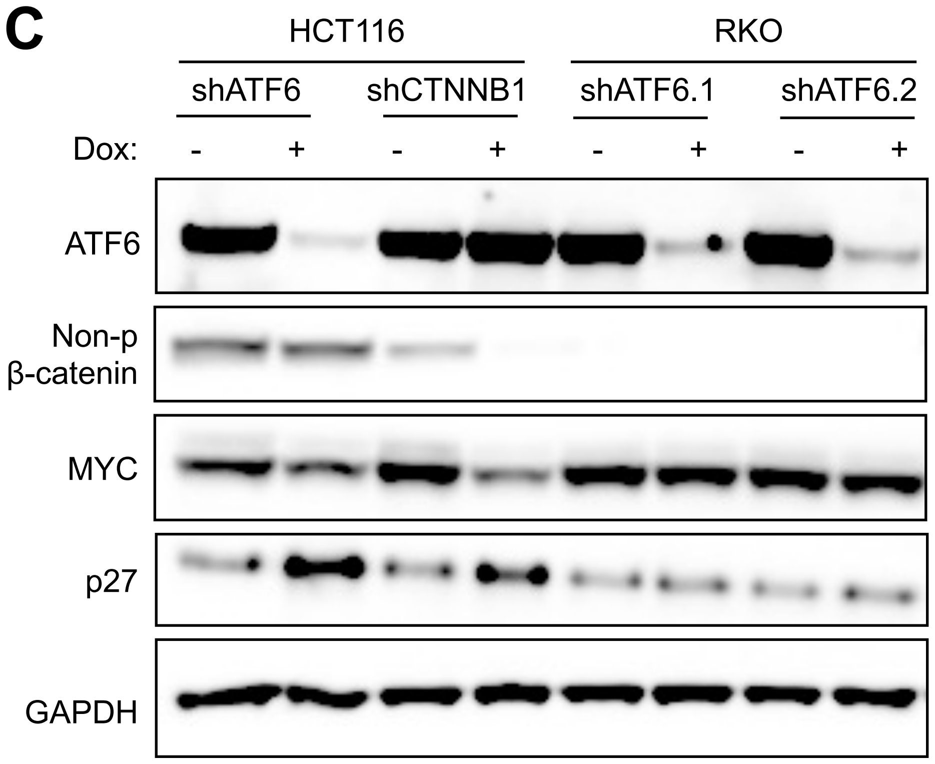

D

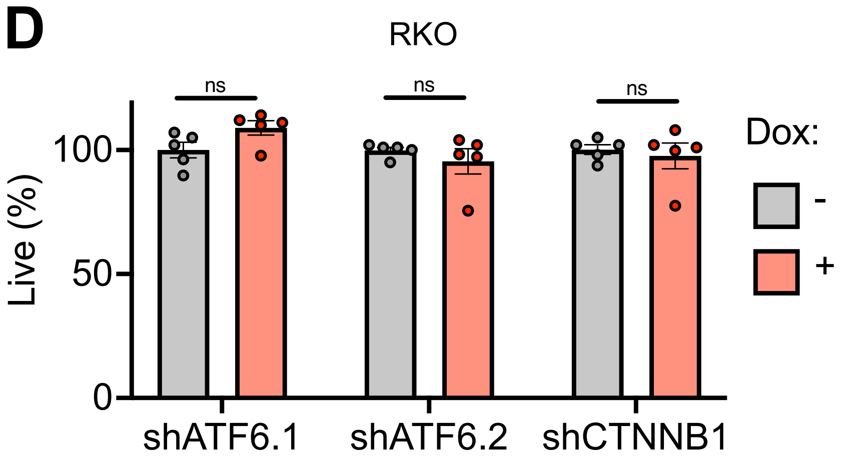

Figure S5: β-catenin knockdown phenocopies ATF6 silencing

(A) Validation of knockdown in HCT116 cells. HCT116 shATF6 cells and HCT116 shCTNNB1 cells were incubated with Dox (0.5 μg/ml) for 2 days and analyzed by IB.

(B) Cell abundance as quantified by RLU of HCT116 shATF6 cells and HCT116 shCTNNB1 cells at specified initial seeding density after 7-day Dox treatment.

(C) HCT116 shATF6 and shCTNNB1 and RKO shATF6 cells incubated with Dox (0.5 μg/ml) for 3 days and analyzed by IB.

(D) Viability of RKO shATF6 and RKO shCTNNB1 cell lines grown in absence or presence of Dox for 7 days (n=5).
