# Supplementary material for: ATF6 Promotes Colorectal Cancer Growth and Stemness by Regulating the Wnt Pathway
Source: Cancer Res Commun. 2024 Oct 21;4(10):2734–55. doi: 10.1158/2767-9764.CRC-24-0268 (PMC11492184; doi:10.1158/2767-9764.CRC-24-0268)
Supplement: Supplementary Figure S6 — Selective inhibition of ATF6 but not IRE1 or PERK attenuates PDM-272 organoid growth [file crc-24-0268_supplementary_figure_s6_supps6.pdf]

Figure S6

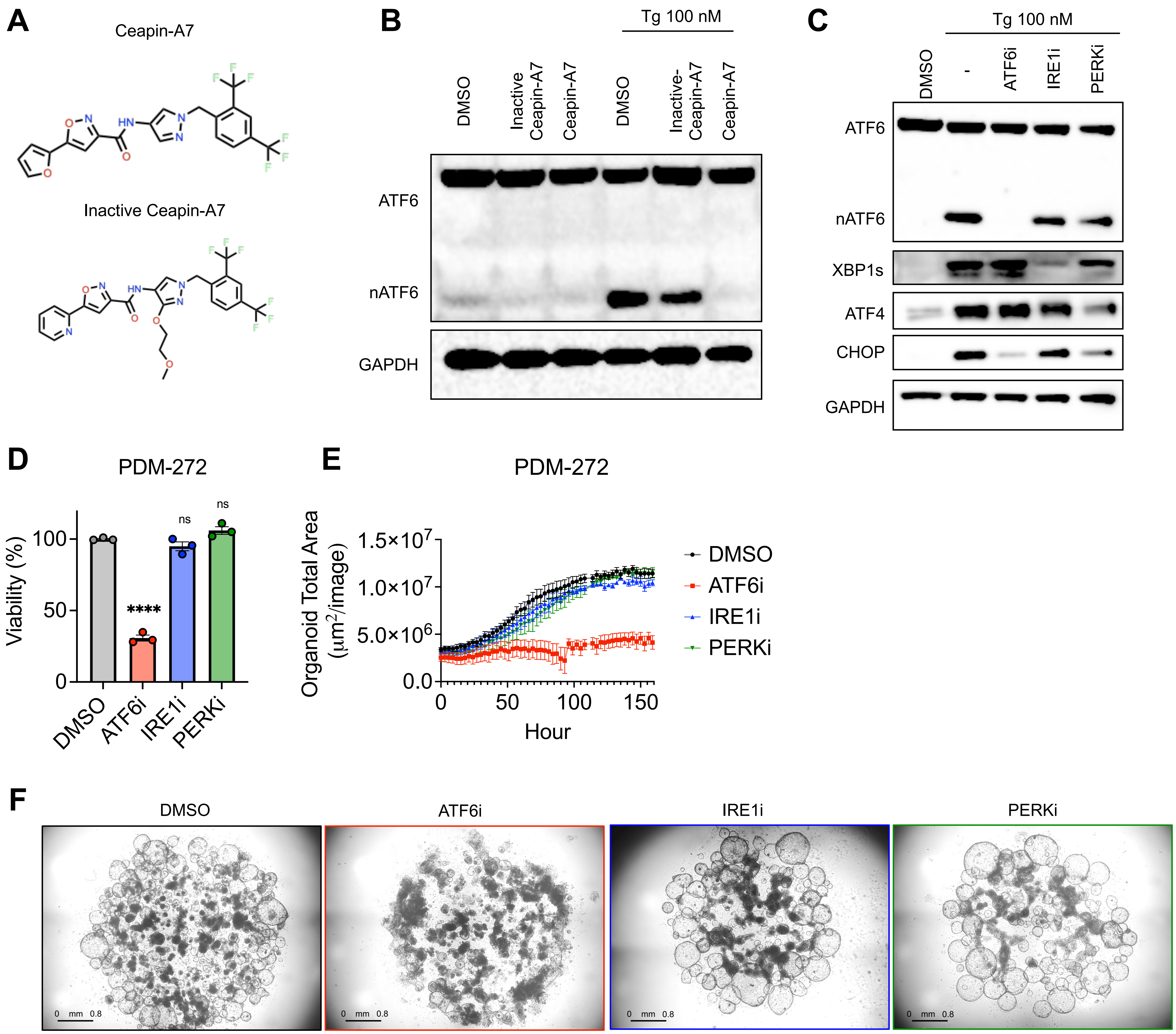
A
Ceapin-A7
Inactive Ceapin-A7
B
Tg 100 nM
DMSO
Inactive Ceapin-A7
Ceapin-A7
DMSO
Inactive-Ceapin-A7
Ceapin-A7
ATF6
nATF6
GAPDH
C
Tg 100 nM
DMSO
-
ATF6i
IRE1i
PERKi
ATF6
nATF6
XBP1s
ATF4
CHOP
GAPDH
D
PDM-272
Viability (%)
100
50
0
****
ns
ns
DMSO
ATF6i
IRE1i
PERKi
E
PDM-272
Organoid Total Area (μm2/image)
1.5×10^7
1.0×10^7
5.0×10^6
0.0
0
50
100
150
Hour
DMSO
ATF6i
IRE1i
PERKi
F
DMSO
ATF6i
IRE1i
PERKi
0 mm 0.8

Figure S6: Selective inhibition of ATF6 but not IRE1 or PERK attenuates PDM-272 organoid growth

(A) Chemical structures of the active and inactive forms of Ceapin-A7.

(B) Validation of Ceapin-A7 and its modified, inactive analog. PDM-272 organoid were treated with or without thapsigargin (Tg) (100 nM) in absence or presence of Inactive Ceapin-A7 (3 μM) or Ceapin-A7 (3 μM) for 4 hours and probed by IB for abundance of nATF6.

(C) Validation of UPR inhibitors. PDM-272 organoid treated with or without Tg (100 nM) in absence or presence of ATF6 inhibition (Ceapin-A7, 3 μM), IRE1 inhibition (4μ8C, 3 μM), or PERK inhibition (AMG PERK 44, 0.5 μM) for 4 hours.

(D) Viability of PDM-272 organoid treated with Ceapin-A7 (ATF6i), 4μ8C (IRE1i), or AMG PERK 44 (PERKi) for 7 days (n=3) as treated in **C**.

(E) PDM-272 organoid confluence as treated in **D**.

(F) Light microscopy images of organoids in **D** after 7-day treatment.
