# Supplementary material for: ATF6 Promotes Colorectal Cancer Growth and Stemness by Regulating the Wnt Pathway
Source: Cancer Res Commun. 2024 Oct 21;4(10):2734–55. doi: 10.1158/2767-9764.CRC-24-0268 (PMC11492184; doi:10.1158/2767-9764.CRC-24-0268)
Supplement: Supplementary Figure S7 — ATF6 inhibition in PDM-272 organoids attenuates cell cycle progression and promotes multilineage intestinal differentiation [file crc-24-0268_supplementary_figure_s7_supps7.pdf]

Figure S7

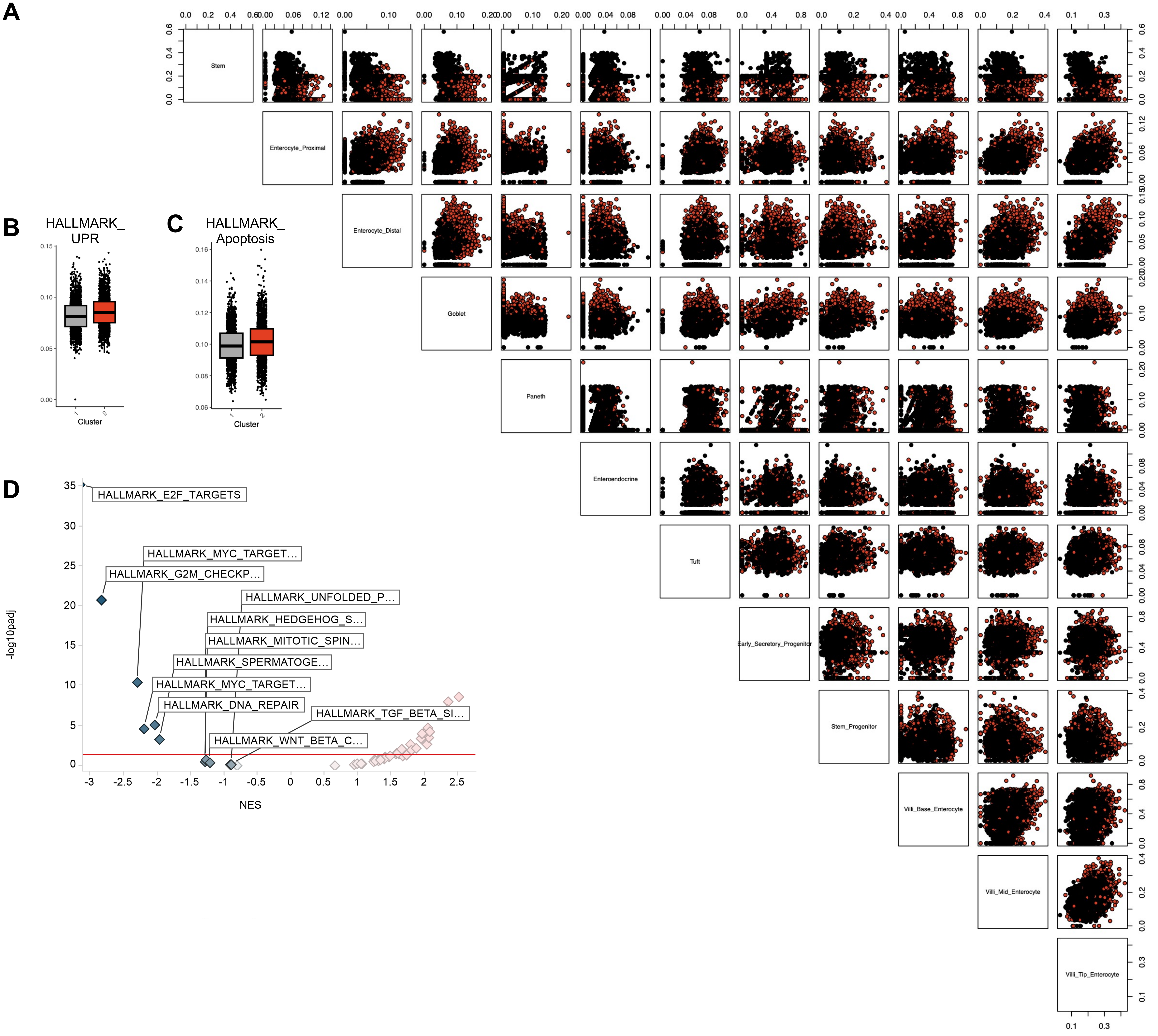
A
Stem
Enterocyte_Proximal
Enterocyte_Distal
Goblet
Paneth
Enteroendocrine
Tuft
Early_Secretory_Progenitor
Stem_Progenitor
Villi_Base_Enterocyte
Villi_Mid_Enterocyte
Villi_Tip_Enterocyte
B
HALLMARK_UPR
Cluster
C
HALLMARK_Apoptosis
Cluster
D
HALLMARK_E2F_TARGETS
HALLMARK_MYC_TARGET…
HALLMARK_G2M_CHECKP…
HALLMARK_UNFOLDED_P…
HALLMARK_HEDGEHOG_S…
HALLMARK_MITOTIC_SPIN…
HALLMARK_SPERMATOGE…
HALLMARK_MYC_TARGET…
HALLMARK_DNA_REPAIR
HALLMARK_TGF_BETA_SI…
HALLMARK_WNT_BETA_C…
-log10padj
NES

Figure S7: ATF6 inhibition in PDM-272 organoids attenuates cell cycle progression and promotes multilineage intestinal differentiation

- **(A)** Scatter plot expression of intestinal cell-lineage gene sets from Cluster 1 (Black) and Cluster 2 (Red) as depicted in **Fig. 6A**.
- **(B)** Hallmark UPR UCell Signature Score of Cluster 1 and Cluster 2 described in **6A**.
- **(C)** Hallmark Apoptosis UCell Signature Score of Cluster 1 and Cluster 2 described in **6A.**
- **(D)** Plot of modulated pathways from the Gene Ontology (GO) analysis of protein expression from proteomics experiment described in **Fig. 6F** based off of Normalized Enrichment Score (NES).
