# Supplementary material for: ATF6 Promotes Colorectal Cancer Growth and Stemness by Regulating the Wnt Pathway
Source: Cancer Res Commun. 2024 Oct 21;4(10):2734–55. doi: 10.1158/2767-9764.CRC-24-0268 (PMC11492184; doi:10.1158/2767-9764.CRC-24-0268)
Supplement: Supplementary Figure S8 — Wnt surrogate restores Wnt-pathway activity and growth of PDM-272 organoids in context of ATF6 inhibition [file crc-24-0268_supplementary_figure_s8_supps8.pdf]

Figure S8

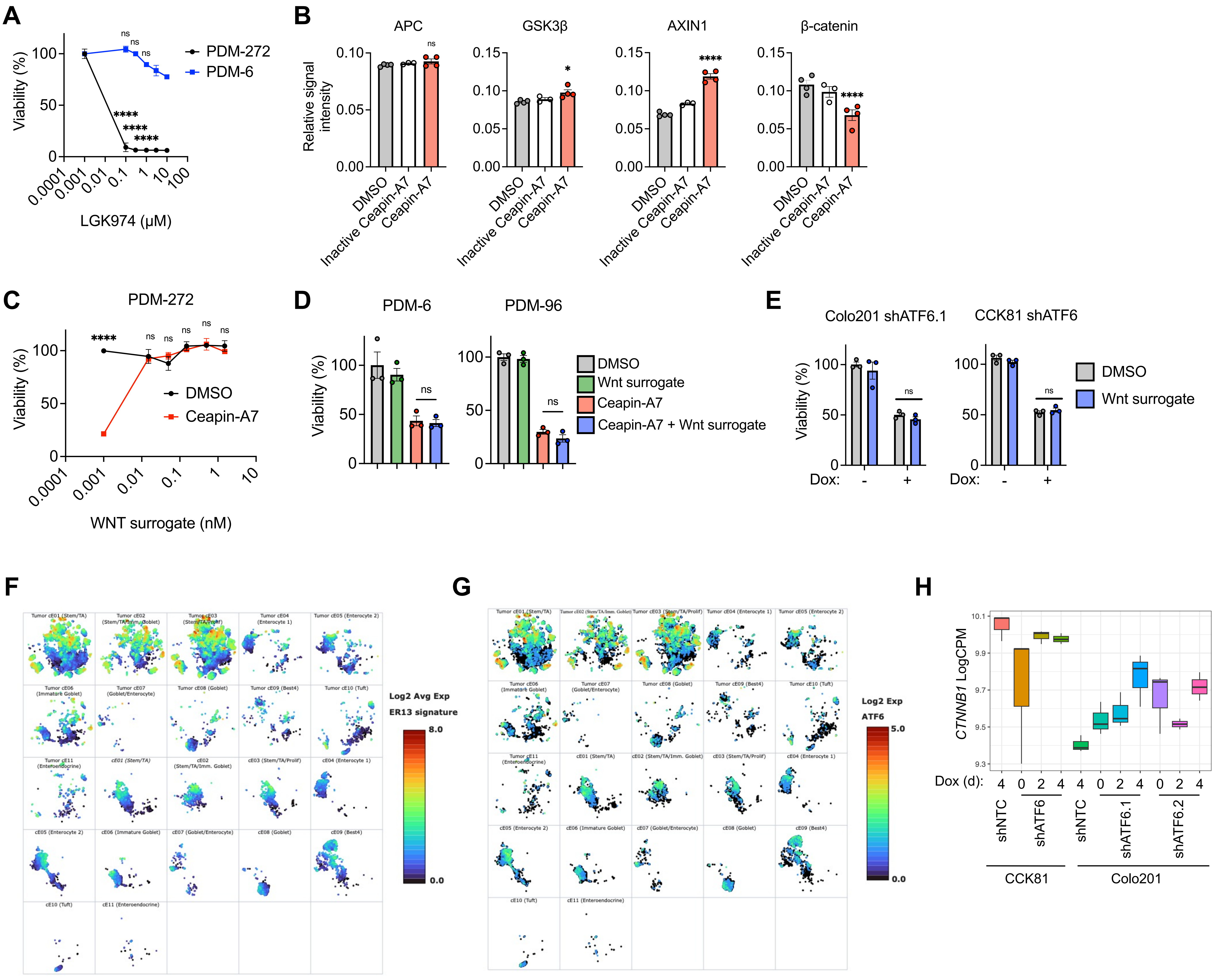
A
Viability (%)
PDM-272
PDM-6
ns
****
LGK974 (μM)
B
APC
GSK3β
AXIN1
β-catenin
Relative signal intensity
DMSO
Inactive Ceapin-A7
Ceapin-A7
ns
*
****
C
PDM-272
Viability (%)
DMSO
Ceapin-A7
WNT surrogate (nM)
D
PDM-6
PDM-96
Viability (%)
DMSO
Wnt surrogate
Ceapin-A7
Ceapin-A7 + Wnt surrogate
E
Colo201 shATF6.1
CCK81 shATF6
Viability (%)
Dox:
DMSO
Wnt surrogate
F
Log2 Avg Exp
ER13 signature
G
Log2 Exp
ATF6
H
CTNNB1 LogCPM
Dox (d):
shNTC
shATF6
shATF6.1
shATF6.2
CCK81
Colo201

Figure S8: Wnt surrogate restores Wnt-pathway activity and growth of PDM-272 organoids in context of ATF6 inhibition

(A) Viability of PDM-272 and PDM-6 organoids treated with Porcupine inhibitor LGK974 at specified concentration for 7 days (n=3).

(B) Effect of ATF6 inhibition on expression of β-catenin destruction complex proteins. Detected abundance of APC, GSK3β, AXIN-1, and β-catenin of PDM-272 organoid as treated and described in **Fig. 6F**.

(C) Viability of PDM-272 after 7-day treatment with or without Ceapin-A7 (3 μM) in the absence or presence of Wnt surrogate at specified concentration (n=3).

(D) Viability of PDM-6, PDM-96 organoids grown for 10 days supplemented with Wnt surrogate (0.01 nM) in absence or presence of Ceapin-A7 (n=3).

(E) Viability of Colo201, CCK81 shATF6 cells grown for 7 days supplemented with Wnt surrogate (0.01 nM) in absence or presence of Dox (0.5 μg/ml) (n=3).

(F) ER13 expression of colon cancer epithelial cell subtypes.

(G) *ATF6* expression of colon cancer epithelial cell subtypes.

(H) Expression of *CTNNB1* in Colo201 and CCK81 shNTC and shATF6 lines upon Dox treatment at specified day.
