# Supplementary material for: ATF6 Promotes Colorectal Cancer Growth and Stemness by Regulating the Wnt Pathway
Source: Cancer Res Commun. 2024 Oct 21;4(10):2734–55. doi: 10.1158/2767-9764.CRC-24-0268 (PMC11492184; doi:10.1158/2767-9764.CRC-24-0268)
Supplement: Supplementary Table S1 — Comparison of ER13 and ER16 gene signatures [file crc-24-0268_supplementary_table_s1_suppst1.pdf]

ER13 Panel (From Shoulders et al, 2013)

Induced by	Gene	Entrez Gene ID
**nATF6**	HSPA5	3309
	HSP90B1	7184
	CALR	811
	PDIA4	9601
	ERO1L	30001
	HERPUD1	9709
	OS9	10956
	SEL1L	6400
**nATF6/XBP1s**	ERDJ3	51726
	ERP57	2923
	PDIA6	10130
	UGGT1	56886
	VCP	7415

ER16 Panel (from Harnoss et al, 2020)

Induced by	Gene	Entrez Gene ID
**IRE1/XBP1s**	DERL1	79139
	DERL2	51009
	DNAJB11	51726
	DNAJB9	4189
	DNAJC10	54431
	DNAJC3	5611
	EDEM1	9695
	EDEM2	55741
	EDEM3	80267
	EIF2AK3	9451
	HERPUD1	9709
	PDIA3	2923
	PDIA6	10130
	SEC61A1	29927
	SERP1	27230
	SYVN1	84447

Supplementary Table S1: Comparison of ER13 and ER16 gene signatures

On left: The ER13 gene signature. ATF6 responsive genes as identified by Shoulders *et al*. that can be induced exclusively by ectopic expression of nuclear ATF6 (nATF6) or also through ectopic express of spliced XBP1 (XBP1s). *On right*: The ER16 gene signature. IRE1 signature gene set as defined by Harnoss *et al.* In red are genes shared between the two subsets.
