# Supplementary material for: ATF6 Promotes Colorectal Cancer Growth and Stemness by Regulating the Wnt Pathway
Source: Cancer Res Commun. 2024 Oct 21;4(10):2734–55. doi: 10.1158/2767-9764.CRC-24-0268 (PMC11492184; doi:10.1158/2767-9764.CRC-24-0268)
Supplement: Supplementary Table S2 — BLAST analysis of ATF6 shRNAs [file crc-24-0268_supplementary_table_s2_suppst2.pdf]

	Description	E value	Max Score	Total Score	Query Cover	Per. ident	Acc. Len	Accession
shATF6-2	Homo sapiens activating transcription factor 6 (ATF6), transcript variant 1, mRNA	0.008	42.1	42.1	100%	100	7470	NM_007348.4
	Homo sapiens activating transcription factor 6 (ATF6), transcript variant 2, mRNA	0.008	42.1	42.1	100%	100	7467	NM_001410890.1
	Homo sapiens cDNA FLJ75428 complete cds, highly similar to Homo sapiens activating transcription factor 6 (ATF6), mRNA	0.008	42.1	42.1	100%	100	2486	AK290498.1
	Homo sapiens activating transcription factor 6, mRNA (cDNA clone IMAGE:4908463), complete cds	0.008	42.1	42.1	100%	100	682	BC014969.1
	Homo sapiens mRNA for activating transcription factor 6 variant protein	0.008	42.1	42.1	100%	100	4368	AB208929.1
	Homo sapiens mRNA for ATF6, complete cds	0.008	42.1	42.1	100%	100	2509	AB015856.1
	Homo sapiens ATF family member ATF6 (ATF6) mRNA, complete cds	0.008	42.1	42.1	100%	100	2474	AF005887.1
	Homo sapiens proline rich membrane anchor 1 (PRIMA1), RefSeqGene on chromosome 14	2.1	34.2	34.2	80%	100	77123	NG_009069.1
	PREDICTED: Homo sapiens potassium voltage-gated channel subfamily D member 3 (KCND3), transcript variant X10, mRNA	32	30.2	30.2	71%	100	24773	XM_054336467.1
	PREDICTED: Homo sapiens potassium voltage-gated channel subfamily D member 3 (KCND3), transcript variant X7, mRNA	32	30.2	30.2	71%	100	10394	XM_011541427.4
	Homo sapiens autophagy related 2B (ATG2B), RefSeqGene on chromosome 14	32	30.2	30.2	71%	100	91147	NG_051091.2
	Homo sapiens GC-rich sequence DNA-binding factor 2 (GCFC2), transcript variant 4, mRNA	32	30.2	30.2	71%	100	4253	NM_001410845.1
	PREDICTED: Homo sapiens uncharacterized LOC105371720 (LOC105371720), transcript variant X3, ncRNA	32	30.2	30.2	71%	100	2070	XR_007087355.1
	PREDICTED: Homo sapiens GC-rich sequence DNA-binding factor 2 (GCFC2), transcript variant X4, mRNA	32	30.2	30.2	71%	100	6173	XM_047445615.1
	PREDICTED: Homo sapiens GC-rich sequence DNA-binding factor 2 (GCFC2), transcript variant X3, mRNA	32	30.2	30.2	71%	100	4142	XM_011533074.4
	PREDICTED: Homo sapiens GC-rich sequence DNA-binding factor 2 (GCFC2), transcript variant X2, mRNA	32	30.2	30.2	71%	100	4277	XM_047445613.1
	PREDICTED: Homo sapiens GC-rich sequence DNA-binding factor 2 (GCFC2), transcript variant X1, mRNA	32	30.2	30.2	71%	100	4391	XM_005264520.5
	PREDICTED: Homo sapiens uncharacterized LOC105371720 (LOC105371720), transcript variant X3, ncRNA	32	30.2	30.2	71%	100	2070	XR_007065696.1
shATF6-4	Homo sapiens activating transcription factor 6 (ATF6), transcript variant 1, mRNA	0.008	42.1	42.1	100%	100	7470	NM_007348.4
	Homo sapiens activating transcription factor 6 (ATF6), transcript variant 2, mRNA	0.008	42.1	42.1	100%	100	7467	NM_001410890.1
	PREDICTED: Homo sapiens activating transcription factor 6 (ATF6), transcript variant X4, mRNA	0.008	42.1	42.1	100%	100	27591	XM_047449542.1
	PREDICTED: Homo sapiens activating transcription factor 6 (ATF6), transcript variant X3, mRNA	0.008	42.1	42.1	100%	100	2187	XM_011509310.3
	Homo sapiens activating transcription factor 6 (ATF6), RefSeqGene on chromosome 1	0.008	42.1	42.1	100%	100	204827	NG_029773.1
	Homo sapiens cDNA FLJ75428 complete cds, highly similar to Homo sapiens activating transcription factor 6 (ATF6), mRNA	0.008	42.1	42.1	100%	100	2486	AK290498.1
	Homo sapiens mRNA for activating transcription factor 6 variant protein	0.008	42.1	42.1	100%	100	4368	AB208929.1
	Homo sapiens activating transcription factor 6, mRNA (cDNA clone IMAGE:6379669), complete cds	0.008	42.1	42.1	100%	100	681	BC071997.1
	Homo sapiens mRNA for ATF6, complete cds	0.008	42.1	42.1	100%	100	2509	AB015856.1
	Homo sapiens ATF family member ATF6 (ATF6) mRNA, complete cds	0.008	42.1	42.1	100%	100	2474	AF005887.1
	**Homo sapiens mitogen-activated protein kinase kinase 3 (MAP2K3), RefSeqGene on chromosome 17**	**0.13**	**38.2**	**38.2**	**90%**	**100**	**37585**	**NG_028256.2**
	**Homo sapiens dual specificity mitogen-activated protein kinase kinase 3 pseudogene (LOC100996792) on chromosome 17**	**0.13**	**38.2**	**38.2**	**90%**	**100**	**10911**	**NG_061548.1**
	Homo sapiens H3K4me1 hESC enhancer GRCh37_chr17:21215299-21215916 (LOC128316625) on chromosome 17	0.13	38.2	38.2	90%	100	818	NG_149229.1
	Homo sapiens calcium voltage-gated channel subunit alpha1 B (CACNA1B), RefSeqGene on chromosome 9	8.1	32.2	32.2	76%	100	253836	NG_042271.1
	Homo sapiens phenylalanyl-tRNA synthetase 2, mitochondrial (FARS2), RefSeqGene on chromosome 6; nuclear gene for mitochondrial product	8.1	32.2	60.5	76%	100	528650	NG_033003.3
	Homo sapiens potassium calcium-activated channel subfamily M alpha 1 (KCNMA1), RefSeqGene on chromosome 10	8.1	32.2	58.5	80%	100	775219	NG_012270.1
	Homo sapiens cDNA FLJ44152 fis, clone THYMU2031341	8.1	32.2	32.2	95%	95	2430	AK126140.1
	Homo sapiens mRNA for FLJ00320 protein	8.1	32.2	32.2	95%	95	5731	AK160376.1
	Homo sapiens taste 1 receptor member 1 (TAS1R1), transcript variant 3, mRNA	32	30.2	30.2	71%	100	2002	NM_177540.3
	Homo sapiens taste 1 receptor member 1 (TAS1R1), transcript variant 2, mRNA	32	30.2	30.2	71%	100	2764	NM_138697.4
	Homo sapiens EPH receptor A3 (EPHA3), RefSeqGene on chromosome 3	32	30.2	30.2	71%	100	381611	NG_023239.2
	Homo sapiens potassium voltage-gated channel subfamily H member 1 (KCNH1), RefSeqGene on chromosome 1	32	30.2	30.2	71%	100	462801	NG_029777.2
	Homo sapiens Rho GTPase activating protein 26 (ARHGAP26), RefSeqGene (LRG_1127) on chromosome 5	32	30.2	30.2	71%	100	465638	NG_016711.2
	Homo sapiens transglutaminase 3 (TGM3), RefSeqGene on chromosome 20	32	30.2	30.2	71%	100	52113	NG_052959.1
	Homo sapiens cholinergic receptor muscarinic 3 (CHRM3), RefSeqGene on chromosome 1	32	30.2	30.2	71%	100	535521	NG_032046.2
	PREDICTED: Homo sapiens taste 1 receptor member 1 (TAS1R1), transcript variant X1, mRNA	32	30.2	30.2	71%	100	3159	XM_011542203.2
	Homo sapiens PHD finger protein 2 pseudogene 2 (PHF2P2) on chromosome 13	32	30.2	30.2	71%	100	18838	NG_032221.2
	PREDICTED: Homo sapiens taste 1 receptor member 1 (TAS1R1), transcript variant X1, mRNA	32	30.2	30.2	71%	100	3159	XM_054338884.1
	Homo sapiens protein kinase C, epsilon (PRKCE) gene, complete cds	32	30.2	30.2	71%	100	544105	JX512458.1
	Homo sapiens cell division cycle associated 4 pseudogene 3 (CDCA4P3) on chromosome 1	32	30.2	30.2	71%	100	890	NG_027199.1
	Homo sapiens isolate SLC3A1-VI-T solute carrier organic anion transporter family member 3A1 (SLCO3A1) gene, complete cds	32	30.2	30.2	71%	100	315834	FJ515841.1
	Homo sapiens ribosomal protein S6 pseudogene 8 (RPS6P8) on chromosome 6	32	30.2	30.2	71%	100	930	NG_011004.1
	Homo sapiens ribosomal protein S6 pseudogene 21 (RPS6P21) on chromosome 12	32	30.2	30.2	71%	100	1002	NG_010836.1
	Homo sapiens cDNA FLJ52254 complete cds, highly similar to Homo sapiens taste receptor, type 1, member 1 (TAS1R1), transcript variant 3, mRNA	32	30.2	30.2	71%	100	1584	AK293499.1
	Homo sapiens taste receptor, type 1, member 1, mRNA (cDNA clone MGC:168128 IMAGE:9020505), complete cds	32	30.2	30.2	71%	100	2760	BC136515.1
	Homo sapiens taste receptor, type 1, member 1, mRNA (cDNA clone MGC:168129 IMAGE:9020506), complete cds	32	30.2	30.2	71%	100	2759	BC136516.1
	Homo sapiens protein kinase C, epsilon (PRKCE) gene, complete cds	32	30.2	30.2	71%	100	539048	EU332867.1
	Homo sapiens cDNA, FLJ96929, highly similar to Homo sapiens taste receptor, type 1, member 1 (TAS1R1), mRNA	32	30.2	30.2	71%	100	2591	AK315805.1
	Homo sapiens cDNA FLJ77460 complete cds, highly similar to Homo sapiens taste receptor, type 1, member 1 (TAS1R1), transcript variant 3, mRNA	32	30.2	30.2	71%	100	1871	AK292014.1
	Homo sapiens transglutaminase 3 (E polypeptide, protein-glutamine-gamma-glutamyltransferase) (TGM3) gene, complete cds	32	30.2	30.2	71%	100	48960	EF102483.1
	TPA: Homo sapiens taste receptor (TAS1R1) mRNA, complete cds	32	30.2	30.2	71%	100	2526	BK000153.1
	Homo sapiens gm148 form C mRNA, partial cds, alternatively spliced	32	30.2	30.2	71%	100	1383	AF387619.1
	Homo sapiens gm148 form B mRNA, complete cds, alternatively spliced	32	30.2	30.2	71%	100	2516	AF387618.1
	Homo sapiens gm148 form A mRNA, complete cds, alternatively spliced	32	30.2	30.2	71%	100	1809	AF387617.1
	Homo sapiens chromosome 5 clone CTB-122H10, complete sequence	32	30.2	30.2	71%	100	145424	AC010602.7
shATF6-6	Homo sapiens activating transcription factor 6 (ATF6), transcript variant 1, mRNA	0.008	42.1	42.1	100%	100	7470	NM_007348.4
	Homo sapiens activating transcription factor 6 (ATF6), transcript variant 2, mRNA	0.008	42.1	42.1	100%	100	7467	NM_001410890.1
	Homo sapiens activating transcription factor 6 (ATF6), RefSeqGene on chromosome 1	0.008	42.1	42.1	100%	100	204827	NG_029773.1
	Homo sapiens mRNA for activating transcription factor 6 variant protein	0.008	42.1	42.1	100%	100	4368	AB208929.1
	Homo sapiens mRNA for ATF6, complete cds	0.008	42.1	42.1	100%	100	2509	AB015856.1
	Homo sapiens ATF family member ATF6 (ATF6) mRNA, complete cds	0.008	42.1	42.1	100%	100	2474	AF005887.1
	Homo sapiens adenylate cyclase 5 (ADCY5), RefSeqGene on chromosome 3	2.1	34.2	34.2	80%	100	173250	NG_033882.1
	Homo sapiens H3K4me1 hESC enhancer GRCh37_chr9:137600147-137600714 (LOC127816657) on chromosome 9	8.1	32.2	32.2	76%	100	768	NG_118147.1
	Homo sapiens caldesmon 1 (CALD1), RefSeqGene on chromosome 7	8.1	32.2	32.2	76%	100	232263	NG_029186.2
	Homo sapiens collagen type V alpha 1 chain (COL5A1), RefSeqGene (LRG_737) on chromosome 9	8.1	32.2	32.2	76%	100	210038	NG_008030.1
	Homo sapiens solute carrier family 25 member 14 (SLC25A14), RefSeqGene on chromosome X; nuclear gene for mitochondrial product	32	30.2	30.2	71%	100	40289	NG_012850.2
	Homo sapiens diacylglycerol kinase beta (DGKB), RefSeqGene on chromosome 7	32	30.2	30.2	71%	100	836729	NG_029494.2
	Homo sapiens solute carrier family 6 member 17 (SLC6A17), RefSeqGene on chromosome 1	32	30.2	30.2	71%	100	58689	NG_051945.1
	PREDICTED: Homo sapiens SLP adaptor and CSK interacting membrane protein (SCIMP), transcript variant X1, mRNA	32	30.2	30.2	71%	100	2217	XM_054316028.1
	Homo sapiens H3K4me1 hESC enhancers GRCh37_chr1:46987892-46988843 and GRCh37_chr1:46988844-46989794 (LOC115801426) on chromosome 1	32	30.2	30.2	71%	100	2147	NG_066084.2
	Homo sapiens serine palmitoyltransferase long chain base subunit 3 (SPTLC3), RefSeqGene on chromosome 20	32	30.2	30.2	71%	100	167132	NG_053155.2
	Homo sapiens histone deacetylase 4 (HDAC4), RefSeqGene on chromosome 2	32	30.2	30.2	71%	100	360482	NG_009235.2
	Homo sapiens SLP adaptor and CSK interacting membrane protein (SCIMP), transcript variant 2, mRNA	32	30.2	30.2	71%	100	2403	NM_001271842.1
	Homo sapiens SLP adaptor and CSK interacting membrane protein (SCIMP), transcript variant 1, mRNA	32	30.2	30.2	71%	100	2424	NM_207103.3
	Homo sapiens SLP adaptor and CSK interacting membrane protein (SCIMP), transcript variant 3, mRNA	32	30.2	30.2	71%	100	2195	NM_001319190.2
	Homo sapiens interferon regulatory factor 4 (IRF4), RefSeqGene on chromosome 6	32	30.2	30.2	90%	94.74	26705	NG_027728.1
	Homo sapiens ras responsive element binding protein 1 (RREB1), RefSeqGene on chromosome 6	32	30.2	30.2	71%	100	151384	NG_016201.1
	Homo sapiens chromosome 17 open reading frame 87, mRNA (cDNA clone MGC:177933 IMAGE:9052916), complete cds	32	30.2	30.2	71%	100	645	BC144395.1
	Homo sapiens DEAH-box helicase 40 (DHX40), transcript variant 1, mRNA	32	30.2	30.2	71%	100	3575	NM_024612.5
	Homo sapiens ZNF594 divergent transcript (ZNF594-DT), transcript variant 1, long non-coding RNA	32	30.2	30.2	71%	100	2172	NR_034082.2
	Homo sapiens mucin 4, cell surface associated (MUC4), RefSeqGene on chromosome 3	32	30.2	30.2	71%	100	72209	NG_053117.1
	Homo sapiens ENAH actin regulator (ENAH), RefSeqGene on chromosome 1	32	30.2	30.2	71%	100	180217	NG_051578.1
	Homo sapiens gamma-aminobutyric acid type A receptor subunit beta2 (GABRB2), RefSeqGene on chromosome 5	32	30.2	30.2	71%	100	266706	NG_047050.1
	Homo sapiens carbonic anhydrase 8 (CA8), RefSeqGene on chromosome 8	32	30.2	30.2	71%	100	102976	NG_023193.2
	PREDICTED: Homo sapiens DEAH-box helicase 40 (DHX40), transcript variant X4, mRNA	32	30.2	30.2	71%	100	3224	XM_047436758.1
	PREDICTED: Homo sapiens DEAH-box helicase 40 (DHX40), transcript variant X3, mRNA	32	30.2	30.2	71%	100	3392	XM_011525253.4
	PREDICTED: Homo sapiens DEAH-box helicase 40 (DHX40), transcript variant X2, mRNA	32	30.2	30.2	71%	100	3407	XM_017025080.3
	PREDICTED: Homo sapiens DEAH-box helicase 40 (DHX40), transcript variant X1, mRNA	32	30.2	30.2	71%	100	3494	XM_006722083.4
	Homo sapiens keratin 26 (KRT26), RefSeqGene on chromosome 17	32	30.2	30.2	71%	100	12922	NG_012487.1

Supplementary Table S2: BLAST analysis of ATF6 shRNAs

Curated list of predicted gene targets of each of the three ATF6 shRNAs (shATF6.2, shATF6.4, shATF6.6) used herein as predicted from Standard Nucleotide BLAST (NCBI) using standard Nucleotide collection. Gene hits were ranked based on e-score. In bold is the single gene found with an e-score <1, i.e., *MAP2K3*. As shown in **Fig. S3I**, Dox treatment of shATF6 lines did not significantly decrease the mRNA expression of *MAP2K3.*
