# Supplementary material for: ATF6 Promotes Colorectal Cancer Growth and Stemness by Regulating the Wnt Pathway
Source: Cancer Res Commun. 2024 Oct 21;4(10):2734–55. doi: 10.1158/2767-9764.CRC-24-0268 (PMC11492184; doi:10.1158/2767-9764.CRC-24-0268)
Supplement: Supplementary Table S3 — Materials Table [file crc-24-0268_supplementary_table_s3_suppst3.pdf]

Materials Table

Reagent or Resource

**IB Antibodies**	**Source**	**Identifier**
ATF6	ProteinTech	66563
GAPDH	CST	2118L
MYC	Abcam	ab32072
p21	CST	2947
p27	BD Bioscience	554069
XBP1 (spliced)	In House	
BiP	CST	3177
ATF4	CST	97038
Non-p-β-catenin	Abcam	ab246504
CHOP	CST	2895
**Flow cytometry reagents**		
Human BD Fc Block	BD	564220
BB700 Rat Anti-Human LGR5	BD	746155
PE-CF594 Rat Anti-Human LGR5	BD	563470
Click-iT EdU Flow Cytometry,	Invitrogen	C10634
Dead Cell Apoptosis Kits with AV for Flow	Invitrogen	V13241
**qPCR Primers**		
ATF6	Thermo Fisher Scientific	Hs00232586_m1
GAPDH	Thermo Fisher Scientific	Hs02786624_g1
MYC	Thermo Fisher Scientific	Hs00153408_m1
AXIN2	Thermo Fisher Scientific	Hs00610344_m1
TCF7	Thermo Fisher Scientific	Hs01556515_m1
LGR5	Thermo Fisher Scientific	Hs00969422_m1
CDKN1A (p21)	Thermo Fisher Scientific	HS00355782_m1
CDCA7	Thermo Fisher Scientific	Hs00230589_m1
CTNNB1 (β-catenin)	Thermo Fisher Scientific	Hs00355045_m1
**Chemicals**		
TransIT-X2 Dynamic Delivery System		
CellTiter-Glo Luminescent Cell Viability Assay	Mirus	MIR 6004
Thapsigargin	Promega	G7571
Doxycycline	In-house	
Ceapin-A7	In-house	
Z-VAD-FMK (zVAD)	In-house	
AMG PERK 44	Selleckchem	S7023
4μ8c	In-house	
Wnt-surrogate Fc Fusion Recombinant Protein	In-house	
Doxycycline Hyclate	Thermo Fisher Scientific	PHG0401
	Sigma	D9891
LGK974	MedChemExpress	HY-17545

Supplementary Table S3: Materials Table
