# Supplementary material for: ATF6 Promotes Colorectal Cancer Growth and Stemness by Regulating the Wnt Pathway
Source: Cancer Res Commun. 2024 Oct 21;4(10):2734–55. doi: 10.1158/2767-9764.CRC-24-0268 (PMC11492184; doi:10.1158/2767-9764.CRC-24-0268)
Supplement: Supplementary Table S4 — Annotation of CRC organoid mutations [file crc-24-0268_supplementary_table_s4_suppst4.pdf]

β-catenin Destruction Complex (Ligand-independent)

Organoid	ATCC Name	APC	AXIN2	AXIN1
PDM-264	HCM-CSHL-0382-C19	APC W421* (Stop Gained) / APC Q1378* (Stop Gained)		
PDM-5	HCM-CSHL-0061-C18	APC S1355Ffs*20 (Frameshift)		
PDM-6	HCM-CSHL-0062-C18	APC E403Kfs*51 (Frameshift) / APC Y1183* (Stop Gained)		
PDM-96	HCM-CSHL-0143-C20	APC R232* (Stop Gained) / APC E1397* (Stop Gained)		
PDM-185	HCM-CSHL-0238-C18	APC R805* (Stop Gained) / APC I1516Yfs*7 (Frameshift)		
PDM-272	HCM-CSHL-0459-C17		AXIN2 A113T (Missense)	AXIN1 R63C (Missense)

Wnt/FZD Ligand interface (Ligand-dependent)

Organoid	ATCC Name	RNF43	ZNRF3
PDM-264	HCM-CSHL-0382-C19		
PDM-5	HCM-CSHL-0061-C18		
PDM-6	HCM-CSHL-0062-C18		
PDM-96	HCM-CSHL-0143-C20		
PDM-185	HCM-CSHL-0238-C18		
PDM-272	HCM-CSHL-0459-C17	RNF43 R132* (Stop Gained)	ZNRF3 R245* (Stop Gained) / ZNRF3 C461* (Stop Gained)

Other oncogenes

Organoid	ATCC Name	KRAS	BRAF	SMARCA4	TP53	ATM	ATR
PDM-264	HCM-CSHL-0382-C19			SMARCA4 R1192H (Missense)	TP53 F113C (Missense)		
PDM-5	HCM-CSHL-0061-C18				TP53 R175H (Missense)		
PDM-6	HCM-CSHL-0062-C18	KRAS G12D (Missense)	BRAF E221* (Stop Gained)				
PDM-96	HCM-CSHL-0143-C20	KRAS G12D (Missense)					
PDM-185	HCM-CSHL-0238-C18	KRAS G12D (Missense)					
PDM-272	HCM-CSHL-0459-C17					ATM V595V (Synonymous)	ATR T1176A (Missense)

Supplementary Table S4: Annotation of CRC organoid mutations

Mutation calls for all CRC organoids based on Public sequencing data from the National Cancer Institute Genomic Data Commons (https://portal.gdc.cancer.gov/cases/30ad857e-34d5-4ae1-8ca1-e962d10d6440?bioId=a14a4f36-9be1-4a6b-83a0-b9e00dcb4115). For RNF43 in PDM-272, 147 of 147 reads showed the R132* Stop-Gain mutation, indicating that it is homozygous.
